# Wnt/Tcf1 pathway restricts embryonic stem cell cycle through activation of the *Ink4/Arf* locus

**DOI:** 10.1371/journal.pgen.1006682

**Published:** 2017-03-27

**Authors:** Anchel De Jaime-Soguero, Francesco Aulicino, Gokhan Ertaylan, Anna Griego, Aniello Cerrato, Aravind Tallam, Antonio del Sol, Maria Pia Cosma, Frederic Lluis

**Affiliations:** 1 KU Leuven Stem Cell Institute, Department of Development and Regeneration, Stem Cell Signalling laboratory, Herestraat 49, Onderwijs en Navorsing 4, Leuven, Belgium; 2 Centre for Genomic Regulation (CRG), The Barcelona Institute of Science and Technology, Dr. Aiguader 88, Barcelona, Spain; 3 Maastricht Centre for Systems Biology (MaCSBio), Maastricht University. Universiteitssingel 60, 6229 ER Maastricht, The Netherlands; 4 Istituto per l'Endocrinologia e l'Oncologia Sperimentale "Gaetano Salvatore", CNR, Napoli, Italy; 5 TWINCORE, Zentrum für Experimentelle und Klinische Infektionsforschung, Hannover, Germany; 6 Luxembourg Centre for Systems Biomedicine (LCSB), University of Luxembourg, 7, Avenue des Hauts-Fourneaux, L-4362 Esch-sur-Alzette, Luxembourg; 7 Universitat Pompeu Fabra (UPF), Dr Aiguader 88, Barcelona, Spain; 8 ICREA, Pg. Lluís Companys 23, Barcelona, Spain; Erasmus MC, NETHERLANDS

## Abstract

Understanding the mechanisms regulating cell cycle, proliferation and potency of pluripotent stem cells guarantees their safe use in the clinic. Embryonic stem cells (ESCs) present a fast cell cycle with a short G1 phase. This is due to the lack of expression of cell cycle inhibitors, which ultimately determines naïve pluripotency by holding back differentiation. The canonical Wnt/β-catenin pathway controls mESC pluripotency via the Wnt-effector Tcf3. However, if the activity of the Wnt/β-catenin controls the cell cycle of mESCs remains unknown. Here we show that the Wnt-effector Tcf1 is recruited to and triggers transcription of the *Ink4/Arf* tumor suppressor locus. Thereby, the activation of the Wnt pathway, a known mitogenic pathway in somatic tissues, restores G1 phase and drastically reduces proliferation of mESCs without perturbing pluripotency. Tcf1, but not Tcf3, is recruited to a palindromic motif enriched in the promoter of cell cycle repressor genes, such as *p15*^*Ink4b*^, *p16*^*Ink4a*^ and *p19*^*Arf*^, which mediate the Wnt-dependent anti-proliferative effect in mESCs. Consistently, ablation of β-catenin or Tcf1 expression impairs Wnt-dependent cell cycle regulation. All together, here we showed that Wnt signaling controls mESC pluripotency and proliferation through non-overlapping functions of distinct Tcf factors.

## Author summary

Studying how to safely expand stem cells in culture is essential for regenerative medicine applications. Hence there is a clear need to decode how the cell cycle of mouse embryonic stem cells (mESCs) is regulated. Tcf3 and Tcf1 belong to the Tcf family of proteins. Tcf/Lef are effectors of the Wnt/β-catenin pathway and Tcf3 controls mESC pluripotency. Here we identified a recruitment site for Tcf1 embedded into a number of cell cycle repressor genes such as *p15*^*Ink4b*^, *p16*^*Ink4a*^ and *p19*^*Arf*^. Tcf1-mediated activation of these genes drastically slows down proliferation of mESCs. In conclusion, here we showed that the Wnt pathway, besides controlling mESC pluripotency via Tcf3, also regulates mESC cell cycle through the recruitment of Tcf1 to the regulatory sites of key cell cycle genes.

## Introduction

Wnt/β-catenin signalling plays an essential role in development, tissue homeostasis and cancer [1]. In addition, activation of the Wnt pathway maintains pluripotency in mouse embryonic stem cells (mESCs) [2] and controls somatic cell reprogramming [3,4]. On the other hand, deregulation or constant activation of Wnt signalling may lead to cancer formation [5].

In the absence of Wnt ligands, β-catenin is recruited by the destruction complex, where it is phosphorylated by GSK3 and subsequently degraded by ubiquitin-mediated proteolysis. Binding of Wnt ligands to their receptors results in the inactivation of the destruction complex, thereby allowing hypophosphorylated β-catenin accumulation [6]. Small molecules such as 6-bromoindirubin-3'-oxime (BIO) [7] or CHIR99021 [8] can also be used to inhibit GSK3 and thus to stabilize β-catenin.

Stabilized β-catenin can enter the nucleus, where it interacts with members of the T cell factor/lymphoid enhancer factor (Tcf/Lef) family. While a single Tcf/Lef gene is found in *Drosophila melanogaster* and *Caenorhabditis elegans*, four Tcf genes, *Tcf1*, *Lef1*, *Tcf3* and *Tcf4* exist in mammals [9]. An important issue that warranted investigation is if the complexity of Tcf factors has also evolved with specialized or redundant functions of the distinct Tcf/Lef factors. Tcf1 and Tcf3 are the most expressed Tcf/Lef factors in pluripotent mESCs [10,11]. Tcf3 acts as a transcriptional repressor of Wnt target genes regulating the pluripotent gene network in mESCs [12,13]. Activation of Wnt/β-catenin pathway reduces the Tcf3 transcriptional repression thereby reinforcing the stability of the core pluripotency network. However, the function of the Wnt transcriptional activator Tcf1 [14] and its target genes in pluripotent mESCs are unknown. Here we show that Tcf/Lef factors regulate distinct target genes showing gene target specialization determining context-specific responses to Wnt signaling.

In somatic stem cells, activation of the canonical Wnt pathway stimulates cell proliferation [6,15] mainly by inducing expression of *c-Myc* and *Cyclin D1* genes [16]. However, even if the mitogenic effects of the Wnt pathway on somatic cells are well known, whether Wnt signalling regulates the cell cycle of pluripotent cells remained unknown.

Pluripotent mESCs, differently to their somatic stem cell counterparts, display a unique and singular cell cycle defined by a fast proliferation rate, characterized by a long S phase and very short G1 and G2 phases [17–20]. The high proliferative rate of mESCs is due to the absence or low expression of Cyclin-Dependent Kinase Inhibitors (CDKIs) such as the Ink4 family members *p15*^*Ink4b*^, *p16*^*Ink4a*^, *p18*^*Ink4c*^ and *p19*^*Ink4d*^, the CIP1/KIP family members *p21*^*Cip1*^, *p27*^*Kip1*^ and *p57*^*Kip2*^ [19,21–24], and *p19*^*Arf*^ [25]. The *Ink4/Arf* locus encodes for *p15*^*Ink4b*^, *p16*^*Ink4a*^ and *p19*^*Arf*^, which are considered strong tumor suppressors. p15^Ink4b^ and p16^Ink4a^, along with the other Ink4 and Cip/Kip family members can slow down cell proliferation by binding to and inhibiting CDK-cyclin complexes. On the other hand, expression of *p19*^Arf^ inhibits the Mdm2 E3 ubiquitin ligase to activate and stabilize p53, which induces expression of the CDKI *p21*^*Cip*^. Therefore, the *Ink4/Arf* locus controls the two main cell cycle inhibitors and tumor suppressor pathways [26,27].

The biological significance of a short G1 phase in mESCs is yet unclear. It has been hypothesized that a short G1 phase might be essential in actively sustaining the pluripotent state. Accordingly, it has been shown that the longer mESCs stay in G1, the more likely they could be subject to signals for cell differentiation [20,28–31]. However, on the other hand, accumulation of mESCs in G1, by inhibition of Cdk2 [32] or by overexpression of p21 or p27 [33] reduces mESC proliferation but does not affect cell pluripotency.

Here, we show that the activation of the canonical Wnt pathway has a dual role in mESCs. Wnt induces the expression of negative regulators of cell cycle; leading to a reduction of cell proliferation and an increase in the number of cells in G1. Furthermore, activation of the Wnt pathway results in the downregulation of some cell differentiation genes, while the expression of pluripotency genes remains unperturbed. The cell cycle effects are dependent on β-catenin and the downstream transcription factor Tcf1 but independent of Tcf3, indicating specialized and non-overlapping functions of Tcf/Lef factors in mESCs. Tcf1 recruitment was enriched at the promoters of cell cycle genes such as in the *Ink4/Arf* locus. Activation of the Wnt pathway induces therefore an increased expression of negative regulators of the cell cycle such as the tumour suppressors *Cdkn2a* (*p16*^*Ink4a*^, *p19*^*Arf*^) and *Cdkn2b* (*p15*^*Ink4b*^). All together our results show that, in contrast to its mitogenic effect in somatic cells, the Wnt/β-catenin pathway triggers an anti-proliferative effect in mESCs via Tcf1 activity.

## Results

### Tcf1 and Tcf3 show non-overlapping DNA binding motifs

We performed comparative gene target analysis of the two most expressed Tcf/Lef factors in mESCs, Tcf1 and Tcf3 [10,14], by chromatin immunoprecipitation combined with DNA sequencing (ChIP-Seq).

**Fig 1:**
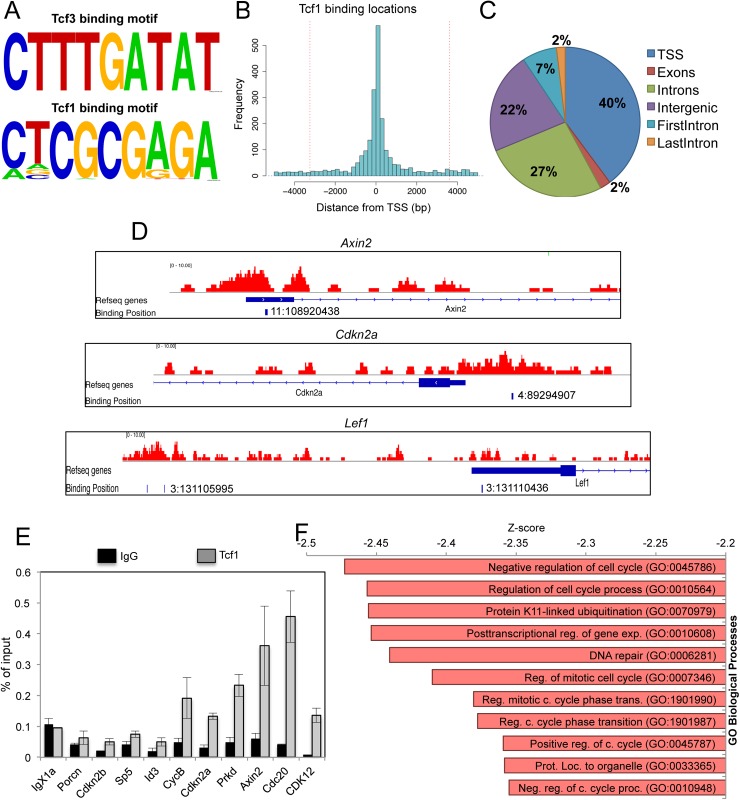
Wnt/Tcf1 pathway controls expression of cell cycle regulators in mESCs. **(A)** Predicted enriched motifs after Tcf3 and Tcf1 ChIP-seq analysis (see Experimental procedures) Tcf1 motif: Hit = 322+/1-, log10 hypergeometric p-value is -99,6. Tcf3 Motif: hit = 71+/0- log10 hypergeometric p-value is -22,4. **(B)** Histogram depicting the distance from Transcriptional Start Site (TSS) of Tcf1 peaks in mESCs. **(C)** Distribution of Tcf1 peaks within the 3 kb region. **(D)** Genome browser snapshot showing the position of Tcf1 peaks near the TSS of the *Ink4/Arf* locus (Cdkn2a), *Axin2* and *Lef1* genes. Genomic coordinates to the binding positions are indicated. **(E)** Independent Tcf1 chromatin immunoprecipitation followed by qRT-PCR for validation of Tcf1 targets genes in mESCs. *IgX1a* is used as negative control. **(F)** Gene Ontology analysis of Tcf1 target genes ranked by z-score (see also S3 Table). The gene set is enriched in cell cycle related genes.

Tcf3 was found to be associated with the canonical Wnt/Tcf DNA binding motif named the Wnt Response Element (WRE: 5′-CTTTGWW-3; W = A or T), as previously reported [34,35] (Fig 1A). We found that Tcf3 binds to ±5 kb of the transcription start site (TSS) of more than 1000 annotated genes (S1 Table). Accordingly with previous reports [13,34], we found that Tcf3 associates with the promoter regions of known Wnt targets (*Axin2*, *Lef1*), pluripotency transcription factor genes (*Oct4*, *Tbx3*, *Nanog*) as well as specific pluripotency miRNAs (*miR302*) (S1 Table) in line with its role as regulator of ESC pluripotency network, of lineage priming and of ESC exit from pluripotency [8].

Interestingly, we found that Tcf1 is recruited to a palindromic DNA binding site (Fig 1A) different from the already described WRE. Most of the peaks (95%) were within 3.5 kb distance from the TSSs (Fig 1B). Among these regions (within ±3.5kb region from TSS), 40% corresponded to promoter regions and 27% to intronic regions (Fig 1C). We found around 1800 annotated genes containing a Tcf1 recruitment site at ±3 kb distance from the TSS. The number of Tcf1 target genes increased up to 2100 when the sequence analysis was extended to ±5 kb from the TSS (S2 Table). Importantly, known Wnt target genes in mESCs such as *Axin2*, *Lef1* and *Cdx1* were identified as Tcf1 targets (Fig 1D and S1A Fig) and some targets were validated by independent ChIP-qRT-PCR (Fig 1E).

Next we followed a reverse strategy to link a list of genes with the transcriptional machinery. We used the Enrichr Analysis Tool [36] to determine which transcription factors regulate the genes that are Tcf1 targets at ±3 kb distance from the TSS (S2 Table). Interestingly, an unknown transcription factor with a “TMTCGCGA” DNA binding sequence was identified as best candidate, which matched the newly identified Tcf1 DNA binding sequence (S1B Fig and S4 Table).

These results show that in the majority of cases Tcf1 and Tcf3 bind to distinct DNA binding motifs in mESCs in different promoter regions (S1C Fig and S5 Table), suggesting that they might control different cellular programs and functions.

### Tcf1 is recruited to the promoters of cell cycle regulator genes such as the INK4 and ARF family members

To explore the biological processes regulated by Tcf1, enriched Gene Ontology (GO) categories associated with Tcf1 target genes were identified and displayed using EnrichNet [37] (S3 Table). Genes associated with the category “Negative Regulators of Cell cycle” (GO:0045786) were highly enriched in Tcf1 targets, indicating that some of the Tcf1 direct target genes might be negative regulators of mESC proliferation (Fig 1F). Analysis of KEGG enriched terms also produced “cell cycle” as the first category of Tcf1-binding genes (S1D Fig).

To date, the crosstalk between signal pathways and transcription factors regulating cell cycle in pluripotent cells is still unexplored. Furthermore, mESCs have a unique cell cycle defined by the absence of the expression of CDK inhibitors [20]. Surprisingly, we found recruitment of Tcf1 to the promoter of genes in the *Ink4/Arf* tumour suppressor locus (also known as *Cdkn2* locus) (Fig 1D and S1E Fig).

**Fig 2:**
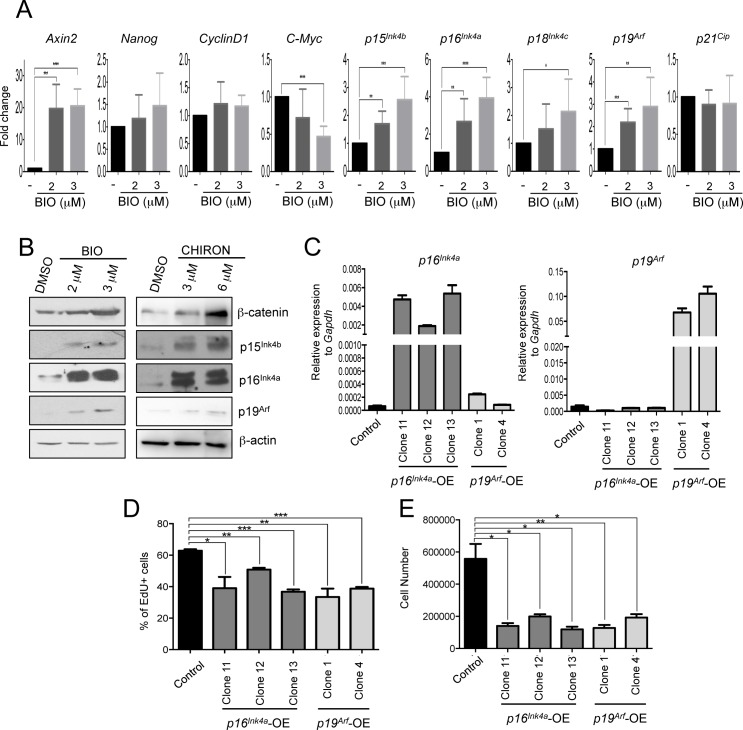
β-catenin stabilization by GSK3 inhibition increases expression of Tcf1 target genes in mESCs. **(A)** Quantitative Real Time PCR (qRT-PCR) for Wnt targets (*Axin2*, *Cyclin D1*, *C-Myc*), stem cell (*Nanog*), and Tcf1 target cell cycle genes (*p16*^*Ink4a*^, *p19*^*Arf*^, *p15*^*Ink4b*^, *p18*^*Ink4c*^ and *p21*^*Cip*^) in control and BIO-treated mESCs for 48h. (n = 6; BIO treated compared to DMSO treated mESCs). **(B)** Representative Western blot of β-catenin, p16^Ink4a^, p19^Arf^, p15^Ink4b^ and β-actin, in 72h BIO and CHIR99021 treated mESCs. **(C)** qRT-PCR of *p16*^*Ink4*a^ and *p19*^*Arf*^ in mESC single clones infected for specific overexpression of p16^Ink4a^ and p19^Arf^. Control cells were infected with empty vector. **(D)** Percentage of EdU positive (EdU+) in control, *p16*^*Ink4a*^ and *p19*^*Arf*^ overexpressing mESCs (p16^Ink4a^-OE, p19^Arf^-OE) 36h after plating (n = 3). Cells were incubated 40’ with EdU before fixation. **(E)** Cell were counted in control, p16^Ink4a^-OE, p19^Arf^-OE mESCs 48h after plating (n = 3). All pooled data are represented as means ± SD. The asterisks indicate statistical significance by two-tailed Student’s t-test analysis (n.s. not significant; * p<0.05; ** p<0.01; ***p<0.001).

To investigate a possible novel function of Wnt/Tcf1 in the regulation of mESC proliferation, we focused on the expression and activity of CDKIs as a new direct target of the Wnt/Tcf1 pathway. We activated the Wnt pathway by treating mESCs with the GSK3 inhibitor BIO. We observed significant upregulation of the transcript level of *p15*^*Ink4b*^, *p16*^*Ink4a*^ and *p19*^*Arf*^ (Fig 2A) after BIO treatment, being *p19*^*Arf*^ the most abundant expressed transcript among them when compared to *Gapdh* levels (S2A Fig). In addition, we also observed an increase in p15^Ink4b^, p16^Ink4a^ and p19^Arf^ protein level along with β-catenin stabilization in mESCs treated with BIO or CHIR99021 for 48h (Fig 2B). Importantly, short-term activation of the pathway did not change the expression of pluripotency markers such as *Nanog*, as expected [3] (Fig 2A). Genes like *c-Myc* and *Cyclin D1*, which are regulated by Wnt signalling and increase the proliferation of a variety of adult stem cells [16], were not upregulated in mESCs after BIO treatment. *Axin2*, a known Wnt target gene, increased, as expected (Fig 2A).

Ink4 family members have a direct role in regulating G1 to S transition [27] and expression of *p19*^Arf^ is known to stabilize p53 [26,27]. Interestingly, in agreement with the increased levels of p19^Arf^, we observed increased levels of p53 together with its downstream target p21^Cip^ in protein nuclear extracts of mESCs treated with BIO for 5 days (S2B Fig) and a reduction of c-Myc protein levels (S2C Fig). Overall, these data indicate that the Tcf1 transcriptional targets belonging to Ink4 and Arf families are upregulated after Wnt pathway activation in mESCs.

Somatic cells slow down their cell cycle and reduce proliferation upon increased expression of any of the genes of the Ink4/Arf locus, such as *p16*^*Ink4a*^ or *p19*^*Arf*^. In addition mESCs are believed to be refractory to the action of some CDKi as *p16*^*Ink4a*^ [21,24]. However, it has recently been shown that an increased expression of p21 or p27 can increase the length mESC G1 and reduce their proliferation [33]. Thus we examined if the Ink4/Arf locus could regulate mESC proliferation. We infected WT mESCs with retroviruses expressing mouse *p16*^*Ink4a*^ or mouse *p19*^*Arf*^ *(*Fig 2C and S2D Fig*)*. Overexpression of *p16*^*Ink4a*^ or *p19*^*Arf*^ did not significantly changed pluripotent marker expression (S2G Fig). Cell proliferation was analysed by EdU staining and cell counting. We observed a reduction of EdU+ cells in all clones overexpressing *p19*^*Arf*^ and in 4 out of 6 clones expressing exogenous *p16*^*Ink4a*^ (Fig 2D and S2E Fig). These results were confirmed by cell counting (Fig 2E and S2F Fig).

All together these data show that cell cycle inhibitors of the Ink4/Arf locus, are transcriptional targets of the Wnt/Tcf1 activation and reduce mESC proliferation.

### The activation of the canonical Wnt pathway inhibits mESC proliferation and increases cells in G1 phase

Activation of the canonical Wnt pathway is necessary to maintain self-renewal and pluripotency of mESCs [2,38]. However, Wnt is also a proliferative signal for intestinal, hair follicle and hematopoietic adult stem cells [39–41], and an oncogenic initiator when aberrantly activated in cancer cells [1,16]. Having identified cell cycle inhibitors as novel Tcf1 target genes in mESCs, we assessed the effect of Wnt pathway activation on mESC morphology, proliferation and cell cycle progression.

**Fig 3:**
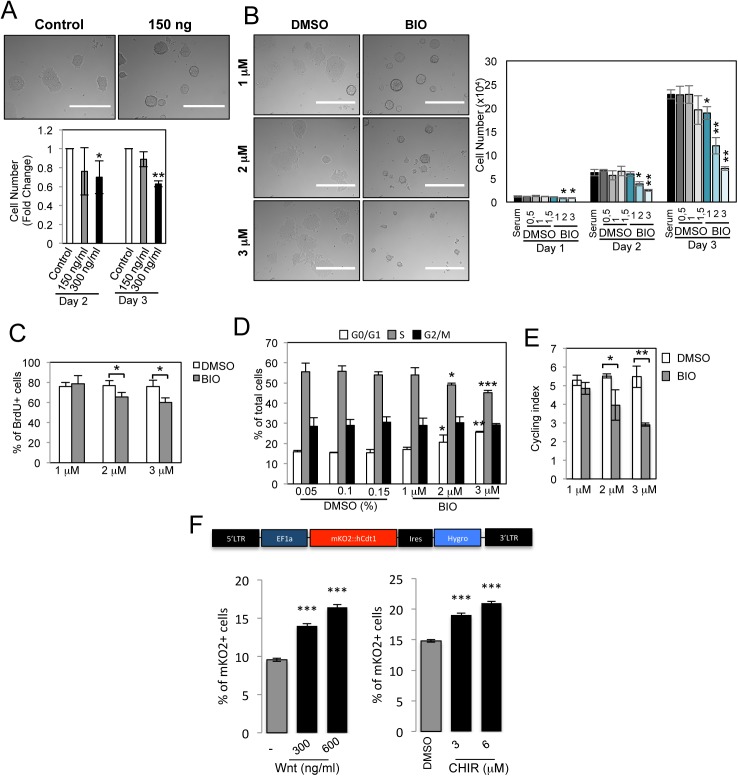
Activation of canonical Wnt pathway in mESCs reduces cell proliferation and increases the number of cells in G1 phase. (*A*) Representative images and quantification of cell number for mESCs treated with purified Wnt3a for 2 and 3 days at the indicated concentrations (n = 3). Scale bar: 400μm. (*B*) Representative images and cell number quantification for mESCs treated with BIO (μM) at indicated concentrations and with DMSO (μl/ml), as control, for 1, 2 and 3 days (n = 3; BIO-treated compared to respective DMSO-treated mESCs). Non-treated (serum) cells are included. Scale bar: 400μm. (*C*) Quantitative representation of number of BrdU positive (BrdU+) mESCs treated with indicated BIO concentrations or DMSO for 72h, as control (n = 3). (*D*) Cell cycle FACS analysis after propidium iodide staining of mESCs treated with BIO or DMSO for 72h (n = 3; BIO-treated compared to respective DMSO-treated mESCs). (*E*) Cycling index (S+G2M/G0G1) of experiments shown in D (n = 3). (*F*) mESCs were modified to express the FUCCI G1 phase reporter. Cells were treated for 72h either with PBS, 300ng/ml or 600ng/ml purified Wnt3a or alternatively with DMSO (0.06%) or 3 and 6 μM of CHIR99021 and analysed by FACS (Three technical replicates). All pooled data are represented as means ± SD. The asterisks indicate statistical significance by two-tailed Student’s t-test analysis (* p<0.05; ** p<0.01; ***p<0.001).

mESCs were cultured under feeder-free conditions with Leukemia inhibitory factor (LIF) and serum and were treated with Wnt3a or with BIO. This successfully stabilized β-catenin (S3A and S3B Fig) and induced increased expression of *Axin2* and *Sp5* target genes (S3C Fig) in a dose-dependent manner. Treatment of mESCs with 0,15% DMSO, used as BIO and CHIR99021 vehicle, did not induced mESC differentiation, as shown by alkaline phosphatase (AP) staining (S3D Fig), neither affected mESC proliferation (Fig 3B). After 3 days of treatment with Wnt3a or BIO, mESCs formed packed colonies with mainly smooth boundaries (Fig 3A and 3B), a characteristic morphology induced by stabilized β-catenin in mESCs [42]. mESC colonies formed in Wnt3a or BIO containing medium were smaller as compared to the colonies formed without these drugs. We observed a significant reduction in total cell number upon 300 ng/ml Wnt3a treatment of mESCs for 48 and 72 hours but not upon 150 ng/ml Wnt3a treatment (Fig 3A). Accordingly, BIO treatment also reduced cell number at concentrations of 2 and 3 μM (Fig 3B) suggesting either Wnt-dependent inhibition of mESC proliferation or Wnt-induced apoptosis.

No significant differences in cell viability and Annexin-V staining were observed in BIO-treated compared to DMSO-treated cells, therefore excluding occurrence of Wnt-induced apoptosis (S3E and S3F Fig). In contrast, after culturing BrdU-labelled mESCs in BIO or DMSO for 72 hours, we observed a reduction of BrdU+ cells in mESCs treated with 2 and 3 μM BIO compared to DMSO-treated cells (Fig 3C). When mESCs were cultured in serum+LIF+DMSO, ~ 55% of cells were in the S phase. However, administration of 2 and 3 μM BIO significantly reduced the number of cells in the S phase, and caused accumulation of cells in G1 (Fig 3D and S3G Fig). The frequency of cells in G2 was similar by comparing BIO-treated and untreated cells. The increased number of cells in G1 was reflected by the significantly lower cycling index, [(S+G2M)/G0G1], of BIO-treated cells compared to untreated cells (Fig 3E). To further validate these results we introduced the Fluorescence Ubiquitination Cell Cycle Indicator (FUCCI) into mESCs [43]. The FUCCI system provides for direct fluorescent visualization of mESCs in G1 phase. As expected, the number of fluorescent mESCs in basal conditions was low in accordance with a very short G1 phase [44]. Interestingly, the number of mESCs activating the G1 phase reporter largely increased upon Wnt pathway activation with Wnt3a or CHIR99021 (Fig 3F and S3H Fig).

All together, these results show that Wnt pathway activation and β-catenin stabilization in mESCs determines an increased number of cells in G1 and a reduced number in the S phase, suggesting an unexpected activity of the canonical Wnt pathway as a negative regulator of proliferation in mESC.

### Pluripotency marker expression is not perturbed upon increased G1 phase in mESCs

**Fig 4:**
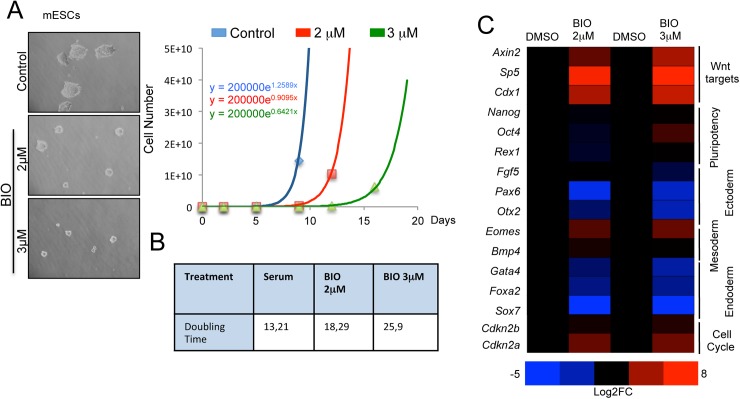
Wnt activated mESCs reduce proliferation without perturbing pluripotency marker expression. **(*A*)** Representative images and quantification of the doubling time of mESCs treated for 8 passages with indicated BIO concentration and DMSO (0.15%) as control. **(*B*)** Population doubling time of mESCs treated with BIO or DMSO for 8 passages. **(*C*)** Heat map of representative qRT-PCR for Wnt targets (*Axin2*, *Sp5*, *Cdx1*), stem cell (*Nanog*, *Oct4*, *Rex1*), ectoderm (*Fgf5*, *Pax6*, *Otx2*), mesoderm (*Eomes*, *Bmp4*) endoderm (*Gata4*, *Foxa2 and Sox7*) and cell cycle (*Cdkn2a*, *Cdkn2b*) marker genes in mESCs treated with BIO or DMSO for 8 passages.

The increased number of mESCs in G1 induced by Wnt activation (Fig 3D and 3F) might be detrimental for pluripotency given that a short G1 phase has been associated with pluripotent state [30,31]. To determine the long-term effects of BIO treatment and Wnt activation on mESC pluripotency and self-renewal we cultured mESCs with 2 and 3 μM BIO for 8 passages and analysed them at population level. At each passage, the cells were counted and the same number of cells was re-plated to calculate the growth rate and the cell doubling time. BIO-treated cells showed reduced cell proliferation during the 8 passages (Fig 4A). The doubling time was increased from 13,2 hours in untreated cells to 18 and 26 hours observed in in 2 and 3 μM BIO-treated cells, respectively (Fig 4B). After 8 passages in BIO-containing medium, the number of mESCs in G1 increased while those in the S phase decreased, as compared to untreated cells (S4A Fig).

At passage 8, pluripotency and differentiation markers were analysed by quantitative RT-PCR (qRT-PCR). No significant changes in the expression level of pluripotent markers (*Oct4*, *Nanog* and *Rex1*) were detected between cells cultured in BIO or DMSO containing media. However, expression of lineage differentiation markers, such as, *Fgf5*, *Pax6*, *Otx2*, *Foxa2 or Sox7* were significantly decreased in BIO-treated cells (Fig 4C). We further confirmed these results by growing 8 independent mESC colonies in DMSO or BIO for 8 passages. mESC clones treated with BIO showed and increased expression of Wnt target genes (*Sp5*, *T*, *Axin2*, *Cdx1* and *Eomes*) [38,45] and equal or reduced expression of many differentiation markers analyzed. Pluripotent markers such as *Nanog* or *Esrrb* did not show differential expression after the treatment. We observed a slight reduction of *Oct4* expression, which has been demonstrated to correlate with a robust pluripotent state [46] and slight *Sox2* increase (S4B Fig).

To investigate whether the effects of BIO on cell cycle length and expression of differentiation markers were reversible, mESCs cultured in BIO containing medium for eight passages were cultured for an additional 8 passages without BIO (No-BIO). After 16 passages (8 BIO + 8 No-BIO) mESCs reverted to a cell cycle and gene expression profile of control cells, i.e. those which had never been treated with BIO (S4C and S4D Fig).

These results indicate that BIO treatment increases the fraction of mESCs in the G1 phase of the cell cycle, thereby significantly increasing the cell doubling time, but it does not affect the expression of pluripotency markers. In addition, while BIO treatment increases the expression of direct Wnt-target genes associated with mesoendoderm differentiation (such as *T* and Eomes), most of analysed differentiation marker genes, which are not direct Wnt target genes, appeared as unchanged or show reduced expression. This result is in agreement with the previously demonstrated Wnt activity to maintain pluripotency and limit lineage priming [13].

### The Wnt pathway inhibits mESC cell cycle via Tcf1/β-catenin and not via Tcf3

**Fig 5:**
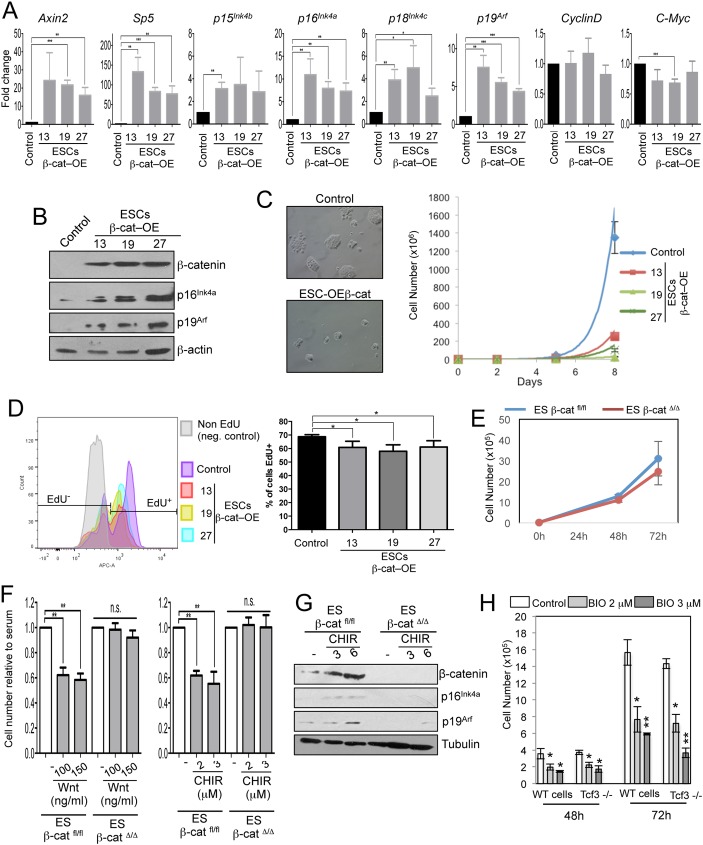
Reduced cell proliferation by Wnt pathway is β-catenin-dependent but Tcf3-independent. **(A)** qRT-PCR for Wnt targets (*Axin2*, *Sp5*, *CyclinD*, *C-Myc*), Tcf1-binding cell cycle genes (*p15*^*Ink4b*^, *p16*^*Ink4a*^, *p18*^*Ink4c*^, *p19*^*Arf*^*)* in control and overexpressing β-catenin clones (ESCs-β-cat–OE clones) (n = 3). **(B)** Representative Western blot of β-catenin, p16^Ink4a^, p19^Arf^ and β-actin in control and ESCs-β-cat–OE clones. **(C)** Representative images and cell growth curve of control mESCs and three overexpressing β-catenin clones (ESCs-β-cat–OE clones 13, 19 and 27) cultured for 3 passages. **(D)** Quantitative representation of number of EdU positive cells (EdU+) in control and ESCs-β-cat–OE clones 36h after plating (n = 4). Cells were incubated 45’ with EdU before fixation. Negative control correspond to control cells non-incubated with EdU. **(E)** Cell number of β-catenin WT (β-catenin^fl/fl^) and Knock-out (β-catenin^Δ/Δ^) mESCs in serum+LIF grown for 3 days. (n = 4). **(F)** Cell number quantification of β-catenin^fl/fl^ and β-catenin^Δ/Δ^ mESCs treated for 48h with purified Wnt3a or with CHIR99021 at indicated concentrations (n = 4). **(G)** Representative Western blot of β-catenin, p16^Ink4a^, p19^Arf^ and β-actin in 48h CHIR99021-treated β-catenin^fl/fl^ and β-catenin^Δ/Δ^ mESCs. **(H)** Cell number quantification of WT and Tcf3-/- ESCs treated with BIO or DMSO (0,15%) at the indicated concentrations for 2 and 3 days (n = 3; BIO-treated compared to respective DMSO-treated mESCs). All pooled data are represented as means ± SD. The asterisks indicate statistical significance by two-tailed Student’s t-test analysis (n.s. not significant; * p<0.05; ** p<0.01; ***p<0.001).

To validate that the results obtained by inhibition of GSK3 were due to β-catenin stabilization and not to other GSK3-dependent cellular substrates [47], we generated three different mESC lines expressing stable β-catenin (ESCs-β-cat–OE). These lines displayed increased levels of stabilized β-catenin protein as well as increased levels of known Wnt/β-catenin target genes such as *Axin2* and *Sp5* (Fig 5A and 5B).

Like Wnt or BIO treated mESCs, β-cat-OE mESC clones formed smaller and more densely packed colonies as compared to WT cells (Fig 5C). Also the number of cells was significantly reduced over three passages (Fig 5C). Furthermore, after culturing both control and β-cat-OE mESC with EdU, we observed a reduction of EdU+ cells in β-cat-OE mESC clones compared to the control cells (Fig 5D). Cell cycle analysis of β-cat-OE mESC clones showed a significant reduction of the number of cells in S phase and in parallel an increase of the cells in G1 phase (S5A Fig). In addition, in ESCs-β-cat-OE clones compared to WT lines we observed a significant increase in transcript and protein levels of the *Ink4* and *Arf* family members (Fig 5A and 5B) and no change in the expression of markers of cell pluripotency (S5B Fig). Thus, constitutive induction of β-catenin is correlated with an increased expression of *Ink4* and *Arf* family members and a reduced mESC proliferation.

To validate and assess if the effects of Wnt activation on mESC cell cycle require β-catenin, we performed β-catenin loss of function experiments by using β-catenin Knock-out (KO) and Knock-down (KD) cells. In basal conditions, control (β-cat^fl/fl^) and β-catenin KO cells (β-cat^Δ/Δ^) [48] showed a comparable proliferation rate (Fig 5E). As expected, the known Wnt targets *Axin2* or *Sp5* were not activated in β-cat^Δ/Δ^ cells after Wnt3a or CHIR99021 treatment (S5C Fig). Interestingly, we observed a decreased proliferation of Wnt3a- or CHIR99021- treated β-cat ^fl/fl^ cells but not of β-cat^Δ/Δ^ cells (Fig 5F).

In addition, short hairpins against different regions of β-catenin were used to generate three distinct mESC lines wherein β-catenin was knocked down (shβcat) (S5E Fig). We treated shControl and shβcat mESCs with 1, 2 and 3 μM BIO and counted the cells after 72 hours by FACS. KD-shβcat cells displayed only a small decrease in cell number when treated with 2 and 3μM BIO while shControl cells showed a drastic reduction (S5E Fig). These results indicate that β-catenin is essential to regulate cell number upon GSK3 inhibition.

Next, we investigated the expression of Ink4 and Arf family members in β-catenin KO and KD cells. In β-catenin KO and KD cells, there was no increase in the expression of *p16*^*Ink4a*^, *p19*^*Ar*f^, *p15*^*Ink4b*^ and *p18*^*Ink4c*^ as compared to respective control cells after GSK3 inhibition (S5D and S5F Fig). In addition, we observed significant upregulation of the protein level of p16^Ink4a^ and p19^Arf^ (Fig 5G) after BIO treatment in control β-cat^fl/fl^ but not in β-cat^Δ/Δ^ cells. All together these results indicate that the expression of the *Ink4/Arf* genes is dependent on β-catenin in mESCs.

As indicated above, Ink4 and Arf family members are targets of Tcf1 but not of Tcf3 in mESCs (S1 Table and S2 Table). Interestingly, following treatment with BIO, the proliferation of mESCs^Tcf3-/-^ cells was comparable to the proliferation of wild type (WT) cells, suggesting that the activity of Tcf3 is not required for the regulation of cell proliferation in response to canonical Wnt signalling (Fig 5H). Moreover, the expression of Ink4 and Arf family members increased in BIO-treated mESCs^Tcf3-/-^ (S5G Fig), excluding that Tcf3 is required for Wnt-dependent cell cycle regulation of mESCs.

All together these data demonstrate that GSK3 inhibition or β-catenin stabilization both transcriptionally regulate the expression of the Ink4 and Arf family members in a Tcf3-independent manner.

### Tcf1 is essential for Wnt-dependent Ink4 and Arf family expression in mESCs

To further investigate the role of Tcf1 in Wnt-dependent cell cycle regulation in mESCs, we used Tcf1 KD cell lines (shTcf1 mESC) [49], wherein Tcf1 RNA levels were reduced by 70% (S6A Fig). Decreased expression of Tcf1 did not impaired the expression of pluripotent markers [49]. We treated shScrmbl and shTcf1 mESCs with BIO for three passages. We observed no difference in the proliferation of shScrmbl and shTcf1 mESCs when cultured with serum+LIF+DMSO. However, addition of BIO induced a reduction in the proliferation of shScrmbl compared to shTcf1 mESCs (S6B Fig). Importantly, upon addition of BIO, *Ink4* and *Arf* genes were not activated in shTcf1 compared to shScrmbl cells (S6A Fig).

We then generated Tcf1 KO mESCs (S6C Fig) using CRISPR/Cas9 technology to further investigate the role of Tcf1 in regulating the expression of Ink4 and Arf family members in mESCs. Deletion of Tcf1 did not affect pluripotent gene expression in mESCs (S6D Fig) as expected [49], and in contrast with another report [50].

**Fig 6:**
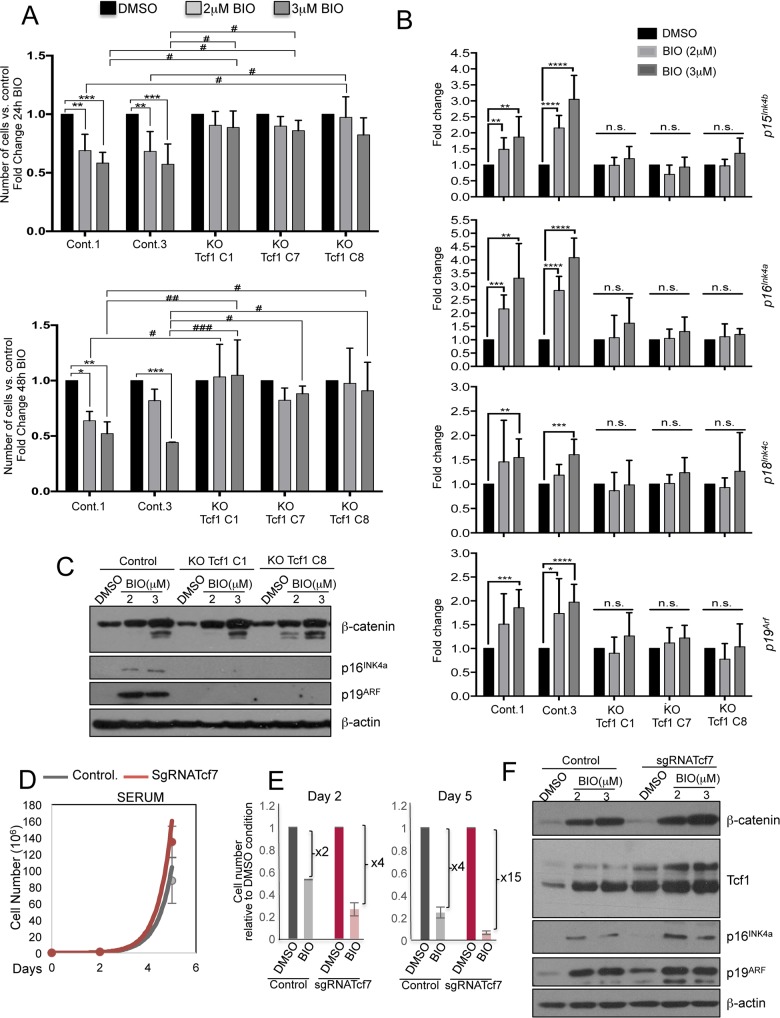
Wnt-dependent cell cycle regulation in mESCs is due to Tcf1 activity. **(A)** Cell number quantification of control and Knock-Out Tcf1 ESC clones treated with indicated BIO concentrations for 24 and 48h (n = 3). The asterisks indicate statistical significance by two-way ANOVA analysis. Statistical significance is shown: (i) between same cell types at different time-points of BIO treatment (as *); and (ii) between different cell types at the same BIO treatment concentration (as #). (n.s. not significant; * or ^#^ p<0.05; ** or ^##^ p<0.01; *** or ^###^ p<0.001). **(B)** qRT-PCR experiment for Tcf1 cell cycle target genes (*p15*^*Ink4b*^, *p16*^*Ink4a*^, *p18*^*Ink4c*^, *p19*^*Arf*^*)* in control and KO Tcf1 clones (clones: C1, C7 and C8) treated with BIO for 48h. (n = 6, two-tailed Student’s t-test analysis, BIO treated cells compared to respective DMSO treated cells.). **(C)** Representative Western blot of β-catenin, p16^Ink4a^, p19^Arf^ and β-actin in 72h BIO-treated WT and Tcf1 KO cells. **(D)** Cell growth curve of dCas9-Vp64-eGFP transduced mESCs, additionally transduced with the empty vector without any sgRNA and sgRNA Tcf1A1 constructs (sgRNATcf7; see S7 Fig) in Serum+LIF conditions (n = 2). **(E)** Cell number quantification of control and Tcf1 overexpressing mESC clones (sgRNATcf7) treated for 2 and 5 days with DMSO (0,15%) and 3 μM of BIO (n = 2). **(*F*)** Representative Western blot of β-catenin, Tcf1, p16^Ink4a^, p19^Arf^ and β-actin in 48h BIO-treated WT and Tcf1-OE cells. All pooled data are represented as means ± SD.

BIO treatment for 24h and 48h reduced the cell number in WT mESCs but not in mESCs^Tcf1-/-^ (Fig 6A). In WT cells, BIO enhanced expression of *Axin2* as well as that of *p15*^*Ink4b*^, *p16*^*Ink4a*^ and *p19*^*Arf*^. In contrast, after BIO treatment of three different mESC^Tcf1-/-^ clones we observed no increase in the expression of Ink4 and Arf family members (Fig 6B and S6E Fig). p16^Ink4a^ and p19^Arf^ protein levels also increased after BIO treatment in WT mESCs but not in mESCs^Tcf1-/-^ (Fig 6C).

Finally, we increased the expression levels of Tcf1 in mESCs using CRISPRa technology [51] (S7 Fig). The three-fold increase of Tcf1 in sgRNATcf7 cells (Fig 6F and S6F Fig) did not have any effect on cell number or expression of pluripotency markers when cells were cultured in serum+LIF (Fig 6D and S6F Fig). However, activation of the Wnt pathway by BIO further increased endogenous Tcf1 expression in sgRNATcf7 and controls (Fig 6F and S6F Fig). The combination of Wnt pathway activation with Tcf1 overexpression induced a strong increase in the expression of *Ink4* and *Arf* family members compared to both DMSO-treated samples as well as to control cells (Fig 6F and S6F Fig). Interestingly, Wnt pathway activation along with Tcf1 overexpression resulted in a strong reduction in cell number (Fig 6E).

### Ink4/Arf knock-down abolishes mESC reduced proliferation induced by Wnt activation

**Fig 7:**
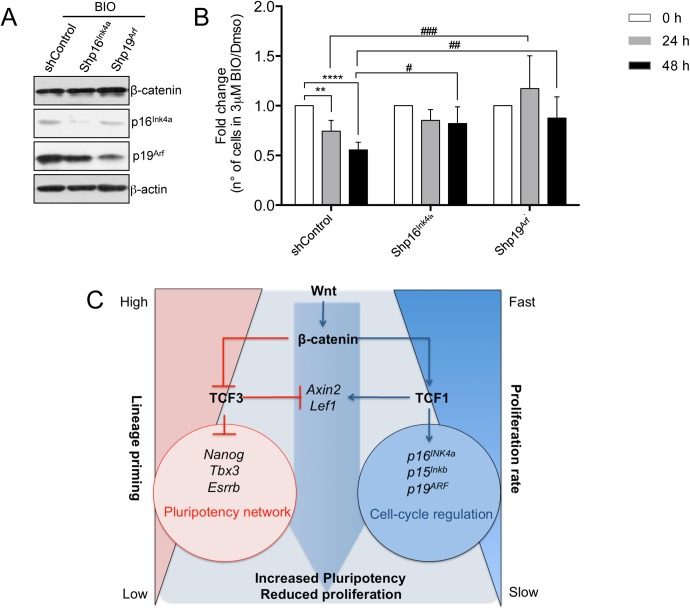
Wnt pathway regulates pluripotency and proliferation in mESCs by non-overlapping activities of Tcf1 and Tc3. **(A)** Representative Western blot of β-catenin, p16^Ink4a^, p19^Arf^ and β-actin in control and mESCs expressing the silencing vectors for p16^Ink4a^ (shp16^Ink4a^) or p19^Arf^ (shp19^Arf^) treated for 48h with BIO 3μM. **(B)** Cell number quantification of control, shp16^Ink4a^ and shp19^Arf^ ESCs treated with 3μM BIO for 24 and 48h (n = 5). Results are shown as fold change of BIO treated cells versus DMSO treated cells. **(C)** Wnt pathway regulates pluripotency network and cell cycle network in mESCs through different Tcf/Lef factors. Activation of the Wnt pathway and stabilization of β-catenin abolishes the repressive activity of Tcf3 on multiple genes of the pluripotency network reducing lineage priming [12,13]. β-catenin stabilization also increases expression of Tcf1 targets as *p16*^*Ink4a*^, *p19*^*Arf*^ and *p15*^*Ink4b*^ which regulate cell cycle and slow down the proliferation rate of ESCs. All pooled data are represented as means ± SD. The asterisks indicate statistical significance by two-way ANOVA analysis. Statistical significance is shown: (i) between same cell type at different time-points after BIO treatment (as #); and (ii) between different cell types at the same time-point (as *). (n.s. not significant; * or ^#^ p<0.05; ** or ^##^ p<0.01; *** or ^###^ p<0.001).

To investigate if the genes encoded by the Ink4/Arf locus, *p16*^*Ink4a*^ and *p19*^*Arf*^, were the main downstream players of the Wnt-dependent reduced proliferation of mESCs, we infected mESCs with retroviruses carrying the KD for *p16*^*Ink4a*^ or *p19*^*Arf*^. Specific KD for *p16*^*Ink4a*^ or *p19*^*Arf*^ reduce their protein levels in mESCs after treatment with BIO (Fig 7A). Cell number was reduced significantly after 24 and 48 hours of BIO treatment in control cells, however no differences in the cell number were observed in BIO treated shp16^*Ink4a*^ and shp19^*Arf*^ mESCs compared to control cells (Fig 7B). A rescue of the proliferative phenotype was observed in p19^*Arf*^ KD infected cells at 24 and 48 hours after BIO treatment, while p16^*Ink4a*^ KD can rescue the phenotype only 48 hours after BIO treatment (Fig 7B).

These results show that the Knock-down of *p16*^*Ink4a*^ or *p19*^*Arf*^ abolish the Wnt-dependent inhibition of cell proliferation in mESCs indicating that *p16*^*Ink4a*^ or *p19*^*Arf*^ are the major players downstream to the Wnt pathway to regulate cell cycle in mESCs.

## Discussion

Our findings show that Tcf1 is required to regulate Wnt-dependent Ink4 and Arf family expression, and this results in the decrease of mESC proliferation. However, reduced expression of Tcf1 has no effect on mESC pluripotency or differentiation [49], in contrast to Tcf3 [12,13], indicating that different Tcf family members mediate divergent functions of the Wnt pathway in mESCs (Fig 7C).

The Wnt pathway has important roles during early development, being activated from the two-cell stage [52] until the pre-implantation blastocyst, and becoming inhibited during post-implantation [38]. In morula stage bovine embryos, ectopic activation of the Wnt pathway inhibits development to the blastocyst stage and this is associated with a significant reduction in total cell number [53]. This observation is in accordance with our findings in mESCs. Given that the activation of the Wnt pathway maintains the pluripotency of mESCs [2], and as we have demonstrated here that it also reduces mESC proliferation, it is reasonable to hypothesise that inhibition of Wnt during the progression of the pre- to post-implantation blastocyst [38] is required to allow cells of the inner cell mass to exit the pluripotent state and start proliferation to produce lineage committed cells.

In the experiments carried out, we activated the Wnt/β-catenin pathway in mESCs using purified Wnt3a and GSK3 specific inhibitors as CHIR99021 and BIO. While these treatments can reduce proliferation of mESCs and increase G1 phase, Wnt3a treatment used at a concentration of 150ng/ml does not induce a significant effect on cell number as also previously reported [14,38]. This is likely due to the fact that high levels of β-catenin stabilization are needed in order to increase p16^Ink4a^ and p19^Arf^ protein levels and therefore to reduce mESC proliferation (S3B Fig). Low levels of purified Wnt3a, as a concentration of 100–150 ng/ml, might be sufficient to maintain mESC pluripotency [14,38] but not enough to induce effects on the cell cycle.

The activity of CDK/cyclin complexes, which are controlled by the expression of CDKI, regulate the transition from one cell cycle phase to another. Contrary to the generally accepted believe that a short G1 phase in mESCs could act as a brake for differentiation [20], we here found that upon Wnt activation, key cell cycle regulators are expressed in mESCs with a consequent increase in the number of cells in G1, which show a prolonged doubling time. Importantly, this is not coupled with a reduced expression of pluripotency genes. We here show that activation of Wnt pathway can increase expression of both *p16*^*Ink4a*^ and *p19*^*Arf*^ at transcriptional and protein level. Previously, it has been shown that mESCs are refractory to p16^Ink4a^ regulation when overexpressed [21,24]. In the present study we overexpressed *p16*^*Ink4a*^ or *p19*^*Arf*^ in mESCs and selected resistant individual clones. We have shown that all the *p19*^*Arf*^ overexpressing clones and four out of the six *p16*^*Ink4a*^ overexpressing clones have a reduced proliferation rate. The fact that two out of six *p16*^*Ink4a*^ clones were resistant to the overexpression of *p16*^*Ink4a*^ and did not reduce their proliferation might suggest that, under certain circumstances, mESCs activate mechanisms to become insensitive to cell cycle regulation as also reported in other studies [21,24].

Finally, the activation of Wnt pathway induces somatic cell proliferation by activating transcription of c-Myc and CyclinD1 [16], while it restricts the cell cycle in pluripotent cells by activating negative cell cycle regulators and reducing c-Myc transcript and protein levels. It is therefore clear that the activation of the Wnt pathway results in opposite outcomes on the proliferation of somatic and pluripotent stem cells. Wnt-induced p19^Arf^ expression in mESCs leads to increased expression of nuclear p53 protein levels. On the other hand, it has been previously shown that p53 binds to the c-Myc promoter and repress its transcription [54]. This might be the reason of the reduced expression of c-Myc in mESCs after Wnt pathway activation. Moreover, it has been recently shown that absence of c-Myc and n-Myc expression induces dormant state in mESCs [55] pointing out the important role of Myc family members on the cell cycle regulation of pluripotent cells.

The identified Tcf1 recruitment DNA motif in mESCs is not a canonical WRE motif. However, it was previously shown that some Tcf1 isoforms could be recruited to alternative C/G-rich DNA binding motifs [9,56]. Furthermore, the Tcf1 motif we identified in mESCs shares the same sequence of KAISO/ZBTB33 in adipocyte-specific promoters [57] or DYRK1A in glioblastoma cell line [58]. Furthermore, Tcf3 in hair follicle cells and Tcf4 in oligodendrocytes were shown to be able to be recruited to the KAISO binding site [35,59] indicating that Tcf/Lef factors can associate with a number of distinct DNA binding domains to regulate gene expression. KAISO was shown to regulate the cell cycle in preadipocytes [57]. Taking into account all these previous observations, it will be important to pursue further investigation on a possible KAISO/Tcf1 coordinated activity in mESCs.

The Wnt pathway acts during evolution starting from metazoans. However, higher organisms present an expanded number of the components of the pathway having four Tcf/Lef members differently from invertebrates that only have one Tcf [9]. Here we show that two different Tcf/Lef factors regulate distinct target genes and control distinct cellular functions in mESCs. Tcf3 regulates self-renewal, potency and lineage priming in mESCs. The expression of Tcf1 does not affect pluripotency. However, Tcf1 regulates mESCs proliferation while Tcf3 does not. All these observations indicate that Tcfs might not be redundant and can regulate context-specific responses of Wnt signalling by activating the expression of different target genes (Fig 7C). Our observations in embryonic stem cells open the path to investigate whether Tcf/Lef factors exert specialized functions also in adult stem cells. Indeed, the Wnt pathway was shown to control both potency and proliferation in hematopoietic and intestinal stem cells, however, whether this is due to the activity of different Tcf factors is not clarified.

Activation of the Wnt pathway as well as transcriptional repression of Tcf1 has been broadly associated with tumour formation [60–62]. Finally, whether the Wnt/Tcf1 pathway also directly controls the regulation of cell cycle and tumor suppressor genes in cancer stem cells will need further investigation. However, it has already been demonstrated that knock-out of the TCF1 gene in mice leads to intestinal tumors as well as highly metastatic thymic lymphomas [60–62], suggesting that Tcf1 is a tumor suppressor gene *per se*. In line with this notion, activation of the Wnt pathway reduces cell proliferation in melanocytes and melanoma [63,64].

## Methods

### Cell culture

R1 and E14Tg2 mouse ESCs were maintained feeder-free on gelatin- (EmbryoMax 0.1% Gelatin Solution, ES-006-B; Millipore) coated plates in DMEM (41965–039 Gibco), 15% fetal bovine serum (Sigma), 2 mM L-glutamine (25030–024; Gibco), 1X minimal essential medium non-essential amino acids (Gibco), penicillin (100 U/ml) /streptomycin (100U/ml) (15140122; Gibco), 100 μM β-Mercaptoethanol (Gibco) and 1,000 U/ml recombinant mouse leukemia inhibitory factor (ESG1107; ESGRO, Chemicon International). mESCs were treated at indicated concentrations to activate the Wnt pathway: purified Wnt3a (315–20; Peprotech); BIO (361550; Calbiochem); CHIR99021 (361571; Calbiochem). BIO and CHIR99021 were resuspended in DMSO (Sigma) at a stock concentration of 2 mM (BIO) and 6 or 10 mM (CHIR99021), Wnt3a (Peprotech) was resuspended at a stock concentration of 50ng/μL following manufacturer instructions.

### Virus infections

For mESC infection, lentiviral particles were produced following the RNA interference Consortium (TRC) instructions for lentiviral particle production and infection in 6-well plates (http://www.broadinstitute.org/rnai/public/). Briefly, 5 ×10^5^ HEK293T cells/well were seeded in 6-well plates. The day after plating, the cells were co-transfected with 1 µg specific lentivirus construct, 750 µg pCMV-dR8.9, and 250 µg pCMV-VSV-G, using Polyfect reagent (Qiagen). The day after transfection, the HEK293T culture medium was substituted with the ESC culture medium. Then 5 ×10^4^ ESCs/well were plated onto gelatin-coated 6-well plates the day before transduction. The lentiviral-containing medium was harvested from HEK293T cells at 48, 72 and 96 h after transfection, filtered, and added to the ESC plates. The day after infection, these mESCs were washed twice in PBS and cultured with normal medium.

Lentiviral constructs for mouse p16^Ink4^ and p19^Arf^ Knock-Down (PIGΔRI-p16^Ink4a^ and PIGΔRI-19^Arf^) were generously provided by Scott Lowe and Manuel Serrano laboratories. Retroviral constructs for mouse p16Ink4a and p19Arf overexpression (pLPC-puro-p16^Ink4a^, pLPC-puro-p19^Arf^) were generously provided by Manuel Serrano laboratory.

### Chromatin immunoprecipitation assay

ChIP was carried out as described in [67]. Briefly, ESCs were trypsinised and crosslinked in 1% formaldehyde at room temperature for 10 min. Crosslinking was quenched with 0.125 M glycine for 5 min. The pelleted cells were lysed in 1 ml ChIP buffer and sonicated in a Bioruptor sonicator (Diagenode) for 10 min. The soluble material was quantified using Bradford assays. To immunoprecipitate the transcription factors, 500 μg protein was used. Antibodies were incubated with the chromatin overnight. The immunocomplexes were recovered with 30 μl protein A or G agarose bead slurries. The immunoprecipitated material was washed three times with low-salt buffer and one time with high-salt buffer. DNA complexes were decrosslinked at 65°C for 3 h, and the DNA was then eluted in 200 μl water using the PCR purification kit (QIAGEN). Two microliters DNA were used for each qPCR reaction. Antibodies used were: Tcf1 (C46C7, Cell Signalling); Tcf3 (sc-8635, Santa Cruz); rabbit IgG (Sigma) and Goat IgG (Santa Cruz).

### Analysis of ChIP-seq results

ChIP-seq reads were barcode-sorted, checked for quality control using Fastqc (http://www.bioinformatics.babraham.ac.uk/projects/fastqc/) and Chance (https://github.com/songlab/chance).

Quality-controlled reads were aligned to the latest mouse genome available (mm10, Genome Reference Consortium GRCm38) using the Bowtie2 version 2.2.0 (http://bowtie-bio.sourceforge.net/bowtie2/index.shtml). Reads with low mapping quality (minimum mapping quality (–q) > = 10) and PCR duplicates were removed using Rmdup from the Samtools (http://samtools.sourceforge.net/) suite. Finally, SAM files were checked and converted to BAM files using Picard (http://broadinstitute.github.io/picard/).

ChIP-seq raw data were submitted to ArrayExpress:

Experiment ArrayExpress accession: E-MTAB-4358

### DNA motif discovery and high-resolution binding-site analysis

Peaks were called using GEM (http://groups.csail.mit.edu/cgs/gem/) [65] high resolution peak calling algorithm with significance level for q-value 1, specified as -log10(q-value) and without the default noise distribution model. We included 1,5 fold enrichment over the control as significant. This allowed us to exclude regions with low signal-to-noise ratios, while including regions that proved reproducible based on ChIP-qPCR even if their overall enrichment was only low to moderate.

Annotated mouse REfSeq genes with a peak at their promoter proximal (±2kb of the transcription start site, TSS) were considered as target. ChIP-seq signal track were visualized by IGV (The Integrative Genomics Viewer).

Gene ontology was analysed using Enrichnet.

### RNA extraction and quantitative PCR detection of mRNA

RNA was extracted and purified using Maxwell Total RNA purification kits (Promega), according to the manufacturer’s instructions.

The cDNA was produced with SuperScript III Reverse Transcriptase kits (Life Technologies) starting from 300 ng to 1 μg mRNA. Real-time quantitative PCR reactions from 8,3 ng of cDNA were set up in triplicate using a DNA SYBR Green I Master Mix (Roche) or Platinum SYBR Green qPCR SuperMix-UDG (Thermoscientific) on a LightCycler machine (Roche) or ViiA 7 Real-Time PCR System (Thermoscientific) respectively. The sequences of the oligonucleotides used in this study are provided on request. Expression levels were normalized to PCR amplification with primers for *Gapdh*.

Statistical analyses were determined by two-tailed Student’s t-test. The 0.05 level of confidence (P<0.05) was accepted for statistical significance.

### Western blot analysis

Cells were harvested and washed twice with PBS. Cell lysis was performed on ice for 25 min, in RIPA buffer (150 mM NaCl, 1% Nonidet P40, 0.5% sodium deoxycholate, 0.1% sodium dodecyl sulphate, 50 mM Tris-HCl, pH 8.0) containing a protease inhibitory cocktail (Roche). Insoluble material was pelleted by centrifugation at 16,000× *g* at 4°C for 3 min. Protein concentrations were determined using the Bradford assay (Bio-Rad). Thirty micrograms extract was mixed with 4× Laemmli buffer (40% glycerol, 240 mM Tris/HCl, pH 6.8, 8% SDS, 0.04% bromophenol blue, 5% β-mercaptoethanol), denatured at 96ºC for 5 minutes, separated by SDS-PAGE, and transferred to nitrocellulose membranes (PROTRAN-Whatman, Schleicher&Schuell). The membranes were blocked with 5% non-fat dry milk in TBS-T for 60 min, incubated with primary antibodies overnight at 4°C, washed three times with TBS-T for 10 min, incubated with the peroxidase-conjugated secondary antibody (1:2000; Amersham Biosciences) in TBS-T with 5% non-fat dry milk for 60 min, and washed three times with TBST for 10 min. Immunoreactive proteins were detected using Supersignal West Dura HRP Detection kits (Pierce). The primary antibodies used were: p16^Ink4a^ (Santa Cruz); p19^Arf^ (Ab80 Abcam); p15^Ink4b^ (Santa Cruz); β-catenin (clone 14, BD Biosciences); p53 (sc-6243 Santa Cruz); p21 (BD Pharmigen); c-Myc (sc-764 Santa Cruz); β-actin (ab8226, abcam).

### Tcf1 knock-out

Two single-guide RNAs (sgRNA+1 [5’-TGCCGCAGCTGGACTCGGG-3’] and sgRNA+1027 [5’-GCTCCGGAGGCCGGTGGGTA-3’]), targeting the first and the third exon of Tcf1 (+1 nd +1027bp from ATG), respectively were cloned separately into pSpCas9(BB)-2A-Puro (PX459). The constructs were co-transfected transiently in mESCs and 24 hours after transfection puromycin selection was applied for an additional 48 hours. Cells were then split and seeded at clonal density. Clones from single cells were manually picked, and analyzed by Western blot for the expression of Tcf1.

pSpCas9(BB)-2A-Puro (PX459) was a gift from Feng Zhang (Addgene plasmid # 48139) [66].

### Tcf1 overexpression

Activation of endogenous Tcf1 promoter was achieved by using a catalytically inactive Cas9 (dCas9) fused to 4 repetition of Vp16 (Vp64) transcriptional activator.

sgRNA were designed using E-crispr online software (http://www.e-crisp.org/) against a region of DNA spanning -400 to -50 bp from TSS of Tcf1.

One sgRNAs was selected: sgRNA Tcf1a1 (5’-GAAGCCTCCAGATTGAGCAA-3’) at -310 from TSS. sgRNA was cloned into pLKO u6 Puro.

Briefly, E14Tg2 mESCs were infected with dCas9Vp674_eGFP and GFP+ cells were FACS-sorted 72hrs after infection to obtain a stable pool expressing dCas9-Vp64. Control cells (infected with dCas9-Vp64+pLKO sgRNA empty vector) and Tcf1 overexpressing cells (infected with dCas9+Vp64+sgRNA Tcf1A1) were selected with puromycin for 72hrs and assessed by qRT-PCR for Tcf1 expression levels.

pLKO.1-puro U6 sgRNA BfuAI large stuffer was a gift from Scot Wolfe (Addgene plasmid # 52628) and dCAS9-VP64_GFP was a gift from Feng Zhang (Addgene plasmid # 61422).

### β-catenin overexpressing mESCs

pCF823, pLenti hEF1a-βcatenin^4A^//SV40-PuroR construct (E[beta]P), containing an unphosphorylatable form of β-catenin (S33A, S37A, T41A and S45A), and vector backbone pRRLSIN-(E(i)P) were used to produce lentivirus particles to infect R1 mESCs. The day after infection, cells were tripsinized and replated to single-cell confluency. Puromycin selection was applied for 4 days and resistant clones were selected and grown individually. Clones displaying high levels of stabilized β-catenin were selected and used for gene expression analysis and growth curve experiments.

E[beta]P was a gift from Roel Nusse (Addgene plasmid # 24313).

### Cell proliferation analysis

For cell counts by hemocytometer, cells were seeded at a uniform density (usually between 25,000 to 40,000 cells per 6 well plate) in the appropriated media. Treatment with Wnt3a, BIO or CHIR99021 was initiated 24 hours after seeding. Cell proliferation of mESCs was assessed by counting the respective cell number in 10μl cell suspension stained with 0,4% trypan blue solution (Sigma) in a Neubauer chamber. For cell counts by FACS cells were trypsinized, diluted in serum containing media and propidium iodide (PI) to detect dead cells. Diluted cells were plated in 96-well plates and counted using FACScanto. For cell growth analysis during several days, mESCs were counted at 48 or 72 hours and replated at the same number for the following days. The total number of cells at each passage was calculated multiplying the number of cells by the product of the previous dilution factors. Exponential growth curves were calculated setting the intercept equal to the number of cells plated at day 0 (*pc*) and the growth rate (*gr*) was used to calculate the doubling time (*dt*).

$$y=pc∙e^{gr∙time};\;dt=\frac{\ln(2)}{gr};$$

Statistical analyses were determined by the unpaired two-tailed Student’s t-test unless indicated in figure legend. The 0.05 level of confidence (P<0.05) was accepted for statistical significance.

### Cell cycle analysis

ES cells were pulse-labeled with 10μM BrdU for 60 min before harvest. Cells were fixed with absolute Ethanol for at least 2 hours. Cells were then washed with PBS+0,5%BSA followed by 15’ incubation of freshly prepared denaturing solution (1ml = 700μl of 0,7%BSA in PBS+ 300μl 25%HCL). After another washing, cells were incubated with (PBS+0,5%BSA+0,5%Tween-20) for 5’. Next, cells were incubated with anti-BrdU antibody conjugated with FITC or with isotype control antibody (BD Pharmingen, 556028) in the dark for 60 minutes. Cells were washed twice with PBS+0,5%BSA and incubated with Propidium Iodide for 30 minutes at RT. Cells were then analyzed by flow-cytometry. ModFit was used as analysis software.

The cycling index was calculated by adding the percentages of cells in S and G2/M phases and dividing them by the percentage in G0/G1 phase (S+G2M)/G0G1.

Statistical analyses were determined by the two-tailed Student’s t-test. The 0.05 level of confidence (P<0.05) was accepted for statistical significance.

### EdU staining

Non-synchronized ES cells were pulse-labeled with 10μM 5-ethynyl-2′-deoxyuridine (EdU, Life Technologies) for 40–60 min. Cells were fixed with 4%PFA for 15 minutes, washed with PBS+2%BSA followed by 15’ permeabilization with PBS+0,5% Triton. Cells were further processed using the Click-IT EdU 555 Imaging kit to reveal EdU incorporation, according to the manufacturer’s instructions, and stained with Propidium Iodide (Life Technologies).

### Cell viability assay

24h after plating, mESC cells were treated with DMSO 0,15% or 3μM BIO or Puromycin 0,4μg/mL. Cells were collected and analyzed every 6h after treatment for 48h. Supernatant and trypsinized cells from each time-point were collected, washed (2x DPBS) and counted. 1x10^6^ cells/mL were stained with 1uL of the BD Horizon Fixable Viability Stain 660 (stock solution) for 12 minutes at room temperature in the dark. Cells were washed twice with 1x DPBS+2%FBS, and fixed (4%PFA) for 15 minutes at room temperature. Viable cells were analyzed in the FACS Canto I. Dot plots and histograms were analyzed by FlowJo v.10 software. As positive technical control of cell death cells were incubated at 65°C for 15 minutes before staining.

### Annexin-V staining

At the indicated incubation time, floating cells were collected together with the supernatant and adherent cells were harvested by trypsinization. Cells were sedimented by centrifugation, counted and 1x10^6^ cells were resuspended in 1 ml of 1x binding buffer (BD Bioscences). Subsequently, 3 μl Annexin-V-APC (BD Biosciences) was added to 100 μl of cell suspension followed by gently vortexing and incubation for 10 min at room temperature in the dark. Thereafter, DAPI was added. Cells were analyzed immediately using a FACS flow cytometer for Annexin-V and DAPI binding. Dot plots and histograms were analyzed by FlowJo v.10 software.

## Supporting information

S1 Fig To respective Fig 1. **(A)** Representative examples of Tcf1-recruitment peaks in *Cdx1* and *Cdkn2c* genes. Genomic coordinates to the binding positions are indicated. **(B)** Enrichr tool was used as reverse analysis method to identify possible transcription factors regulating Tcf1 gene targets (from S2 Table). A transcription factor, binding to TMTCGCGANR DNA motif that matches with Tcf1 DNA binding motif (Fig 1A) was predicted as the highest scored candidate (for the complete analysis see also S4 Table). **(C)** Comparison of Tcf1 and Tcf3 targets genes localized at 3 kb from TSS (S1 Table and S2 Table) **(D)** Table of KEGG enriched terms ranked for adjusted P-value. **(E)** Gene Ontology table showing the list of Tcf1 target genes included in the first two most relevant categories: Negative Regulation of cell cycle (GO:0045786) and Regulation of cell cycle process (GO:0010564) (for the complete list of Tcf1 target genes present in all Gene Ontology categories see also S3 Table).
(TIF)

S2 Fig To respective Fig 2. **(A)** qRT-PCR for Tcf1-recruited cell cycle genes (*p15*^*Ink4b*^, *p16*^*Ink4a*^ and *p19*^*Arf*^*)* in control and BIO treated mESCs for 48h (n = 3). Results are presented as relative expression to *Gapdh* in order to visualize the expression levels of each transcript. **(B)** Representative Western blot of nuclear extracts of p53, p21^Cip^ and Histone 3 (H3) as loading control in 6 days BIO treated WT mESCs. **(C)** Representative Western blot of total c-Myc and β-actin in control and BIO treated mESCs. **(D)** qRT-PCR of *p16*^*Ink4a*^ and *p19*^*Arf*^ in mESCs single clones infected for specific overexpression of *p16*^*Ink4a*^ and *p19*^*Arf*^. Control cells were infected with empty vector. The clones are generated from a second independent infection corresponding to Fig 2C–2E. **(E)** Quantitative representation of number of EdU positive cells (EdU+) in control, p16^Ink4a^ and p19^Arf^ overexpressing mESCs (p16Ink4-OE, p19Arf-OE) 36h after plating (n = 3). Cells were incubated 40’ with EdU before fixation. **(F)** Cell counting quantification of control, p16^Ink4a^-OE, p19^Arf^-OE mESCs 48h after plating (n = 3). **(G)** qRT-PCR for pluripotent stem cell markers (*Esrrb*, *Nanog*, *Oct4*, *Sox2*, *Rex1 and Klf4*) in control (2 clones), p16^Ink4a^-OE (6 clones) and p19^Arf^-OE (3 clones) mESCs.
All pooled data are represented as means ± SD. The asterisks indicate statistical significance by two-tailed Student’s t-test analysis (n.s. not significant; * p<0.05; ** p<0.01; ***p<0.001).
(TIF)

S3 Fig To respective Fig 3. **(A)** Representative Western blots of total β-catenin and β-actin in mESCs treated with Wnt3a or BIO at indicated concentrations for 48h. **(B)** Representative Western blots of total β-catenin, p16^Ink4a^, p19^Arf^ and β-actin in mESCs treated with Wnt3a, CHIR99021 and BIO for 48h at the indicated concentrations. **(C)** qRT-PCR for Wnt target *genes (Axin2*, *Sp5)* in Wnt3a or BIO treated mESCs at indicated concentrations for 48h (n = 4). **(D)** Quantitative representation of the number of colonies stained for Alkaline Phosphatase (AP) in untreated, DMSO and BIO treated mESCs. **(E)** Quantitative representation of live cells by FACS viability assay in time course of DMSO and BIO treated cells (n = 3; mean± S.E.M.). Puromycin was used as experimental positive control of cell death. For positive technical control of cell death, cells were treated with heat shock for 15’. **(F)** Quantitative representation of Annexin V positive (AnnexinV+) mESCs treated with indicated concentrations of BIO or DMSO for 6, 12, 24 and 48h. Puromycin was used as experimental positive control of cell death. **(*G*)** Representative cell cycle FACS analysis of propidium iodide stained mESCs treated with the indicated BIO concentrations for 72h. **(*H*)** Representative FACS analysis of mKO2-hCdt1 mESCs treated with indicated concentrations of Wnt3a and CHIR99021 for 72h.
All pooled data are represented as means ± SD unless specifically indicated. The asterisks indicate statistical significance by two-tailed Student’s t-test analysis (* p<0.05; ** p<0.01; ***p<0.001).
(TIF)

S4 Fig To respective Fig 4. **(A)** Cell cycle quantification by FACS analysis of propidium iodide stained mESCs treated for 8 passages with BIO or with DMSO (n = 3; BIO-treated compared to DMSO-treated mESCs). **(B)** qRT-PCR for Wnt targets (*Axin2*, *Sp5*, *T*, *Cdx1*, *Eomes*), stem cell (*Nanog*, *Oct4*, *Sox2*, *Esrrb*), ectoderm (*Fgf5*, *Pax6*, *Otx2*), mesoderm (*Gossecoid*) endoderm (*Gata4*, *Sox17 and Sox7*) marker genes in independent mESCs clones treated with DMSO (0,15%) and BIO (3μM) for 8 passages. **(C)** Cell cycle quantification by FACS analysis of propidium iodide stained mESCs treated for 8 passages with BIO at the indicated concentrations + 8 passages in serum+LIF without BIO. **(D)** qRT-PCR of Wnt targets (*Axin2*, *Sp5*), stem cell (*Nanog*, *Oct4*), ectoderm (*Fgf5*, *Pax6*, *Otx2*), mesoderm (*Bmp4*) and endoderm (*Gata4*, *Foxa2 and Sox7*) genes in mESCs treated for 8 passages with BIO followed or not by additional 8 passages in serum+LIF medium without BIO.
All pooled data are represented as means ± SD. The asterisks indicate statistical significance by two-tailed Student’s t-test analysis (* p<0.05; ** p<0.01; ***p<0.001).
(TIF)

S5 Fig To respective Fig 5. **(A)** Cell cycle FACS analysis after propidium iodide and EdU staining of control and ESCs-β-cat–OE clones (n = 4). **(B)** qRT-PCR of pluripotent stem cell markers in control and ESCs-β-cat–OE clones (n = 3). **(C)** qRT-PCR for Wnt targets (*Axin2*, *Sp5*) in control (β-catenin^fl/fl^) and β-catenin KO (β-catenin^Δ/Δ^) mESCs treated with Wnt3a or CHIR99021 at indicated concentrations (n = 3; treated cells compared to respective DMSO-treated mESCs). (**D**) qRT-PCR of cell cycle and Wnt targets in control (β-catenin^fl/fl^) and β-catenin KO (β-catenin^Δ/Δ^) mESCs treated for 48h at the indicated CHIR99021 (μM) concentrations (n = 2). **(E)** Cell number quantification of control mESCs and of three different pools of β-catenin silenced (shβcat) mESCs treated for 72h with indicated BIO concentrations (n = 3). **(F)** Heat map of representative qRT-PCR experiments for β-catenin (*Ctnnb1*), Wnt targets (*Axin2*, *Sp5*, *Cdx1*), and Tcf1 binding cell cycle genes (*p15*^*Ink4b*^, *Cdkn2a*, *p18*^*Ink4c*^ and *p19*^*Arf*^) in control and in three different β-catenin silenced mESC pools (shβcat pool 1, 2 and 3) treated for 48h at the indicated BIO concentrations. **(G)** qRT-PCR for Tcf1 binding cell cycle genes (*p15*^*Ink4b*^, *p16*^*Ink4a*^, *p19*^*Arf*^*)* in untreated or BIO-treated mESCs Tcf3^-/-^ at the indicated concentrations for 48h (n = 2).
All pooled data are represented as means ± SD. The asterisks indicate statistical significance by two-tailed Student’s t-test analysis (n.s. not significant; * p<0.05; ** p<0.01; ***p<0.001).
(TIF)

S6 Fig To respective Fig 6. **(A)** qRT-PCR for *Tcf1*, Wnt target (*Axin2*) and Tcf1 binding cell cycle genes (*p15*^*Ink4b*^, *p18*^*Ink4c*^, *Cdkn2a)* in shScrmbl and shTcf1 mESCs treated at the indicated BIO concentration for 48h (n = 2). **(B)** Growth curve of shScrmbl and shTcf1 mESCs cultured for 3 passages and treated with the indicated concentrations of BIO (n = 2). **(C)** Representative Western blot of Tcf1 and β-actin in control and KO Tcf1 mESC clones generated by CRISPR/Cas9. **(D)** qRT-PCR for pluripotent markers (*Oct4*, *Sox2* and *Nanog*) in control (6 mESCs clones) and KO-Tcf1 (13 mESCs clones). **(E)** qRT-PCR for Wnt target genes (*Axin2 and Tcf1)* in control and KO Tcf1 clones treated with BIO for 48h (n = 6; BIO-treated compared to respective DMSO-treated mESCs). **(F)** qRT-PCR for stem cell (*Nanog*, *Rex1*), Wnt targets (*Tcf1* and *Axin2)* and Tcf1 cell cycle target genes (*p15*^*Ink4b*^, *p16*^*Ink4a*^, *p18*^*Ink4c*^, *p19*^*Arf*^*)* in control and Tcf1 overexpressing pool (sgRNATcf7) (one representative experiment).
All pooled data are represented as means ± SD. The asterisks indicate statistical significance by two-tailed Student’s t-test analysis (n.s. not significant; * p<0.05; ** p<0.01; ***p<0.001).
(TIF)

S7 Fig to respective Fig 6. Schematic view of CRISPR/dCas9 method used to overexpress endogenous Tcf1. Two different sgRNAs targeting Tcf1 promoter region (108 and 314 bp from TSS of Tcf1) were used to allow binding of Cas9 fused with Vp64 transactivator domain to Tcf1 promoter in order to increase Tcf1 endogenous expression.
(TIF)

S1 Table TCF3 TSS occupancy from ChIP-seq data. (XLSX)

S2 Table TCF1 TSS occupancy from ChIP-seq data. (XLSX)

S3 Table Functional analysis (G) and KEGG). (XLSX)

S4 Table Reverse analysis-Genome Browser PMWs. (XLSX)

S5 Table Common Target-GenesTCF1& TCF3-within-3k- TSS. (XLSX)
