# Supplementary figures and images for: Wnt/Tcf1 pathway restricts embryonic stem cell cycle through activation of the Ink4/Arf locus
Source: PLoS Genet. 2017 Mar 27;13(3):e1006682. doi: 10.1371/journal.pgen.1006682 (PMC5386305; doi:10.1371/journal.pgen.1006682)

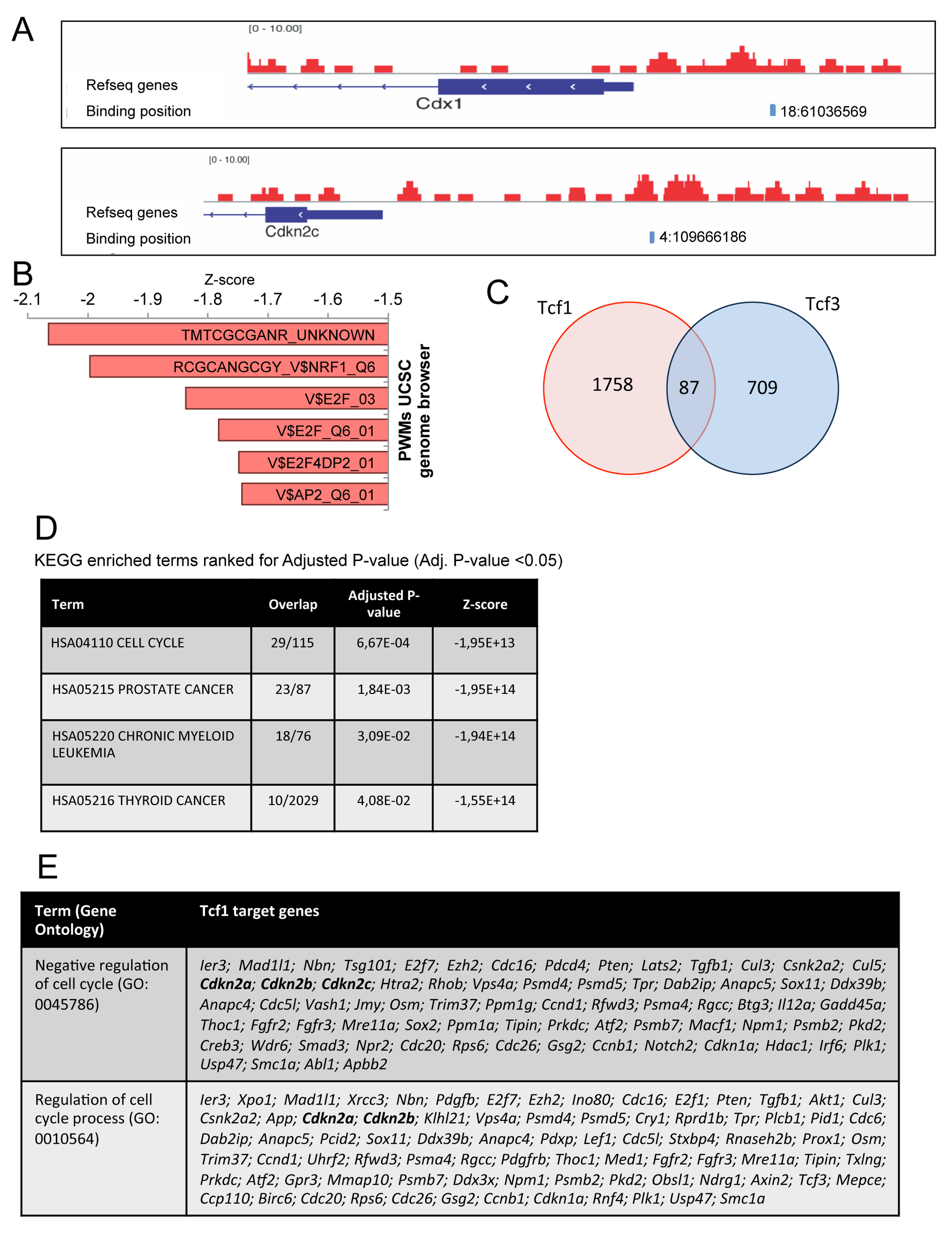

Supplement: S1 Fig — (A) Representative examples of Tcf1-recruitment peaks in Cdx1 and Cdkn2c genes. Genomic coordinates to the binding positions are indicated. (B) Enrichr tool was used as reverse analysis method to identify possible transcription factors regulating Tcf1 gene targets (from S2 Table). A transcription factor, binding to TMTCGCGANR DNA motif that matches with Tcf1 DNA binding motif (Fig 1A) was predicted as the highest scored candidate (for the complete analysis see also S4 Table). (C) Comparison of Tcf1 and Tcf3 targets genes localized at 3 kb from TSS (S1 Table and S2 Table) (D) Table of KEGG enriched terms ranked for adjusted P-value. (E) Gene Ontology table showing the list of Tcf1 target genes included in the first two most relevant categories: Negative Regulation of cell cycle (GO:0045786) and Regulation of cell cycle process (GO:0010564) (for the complete list of Tcf1 target genes present in all Gene Ontology categories see also S3 Table). (TIF) [file pgen.1006682.s001.tif]

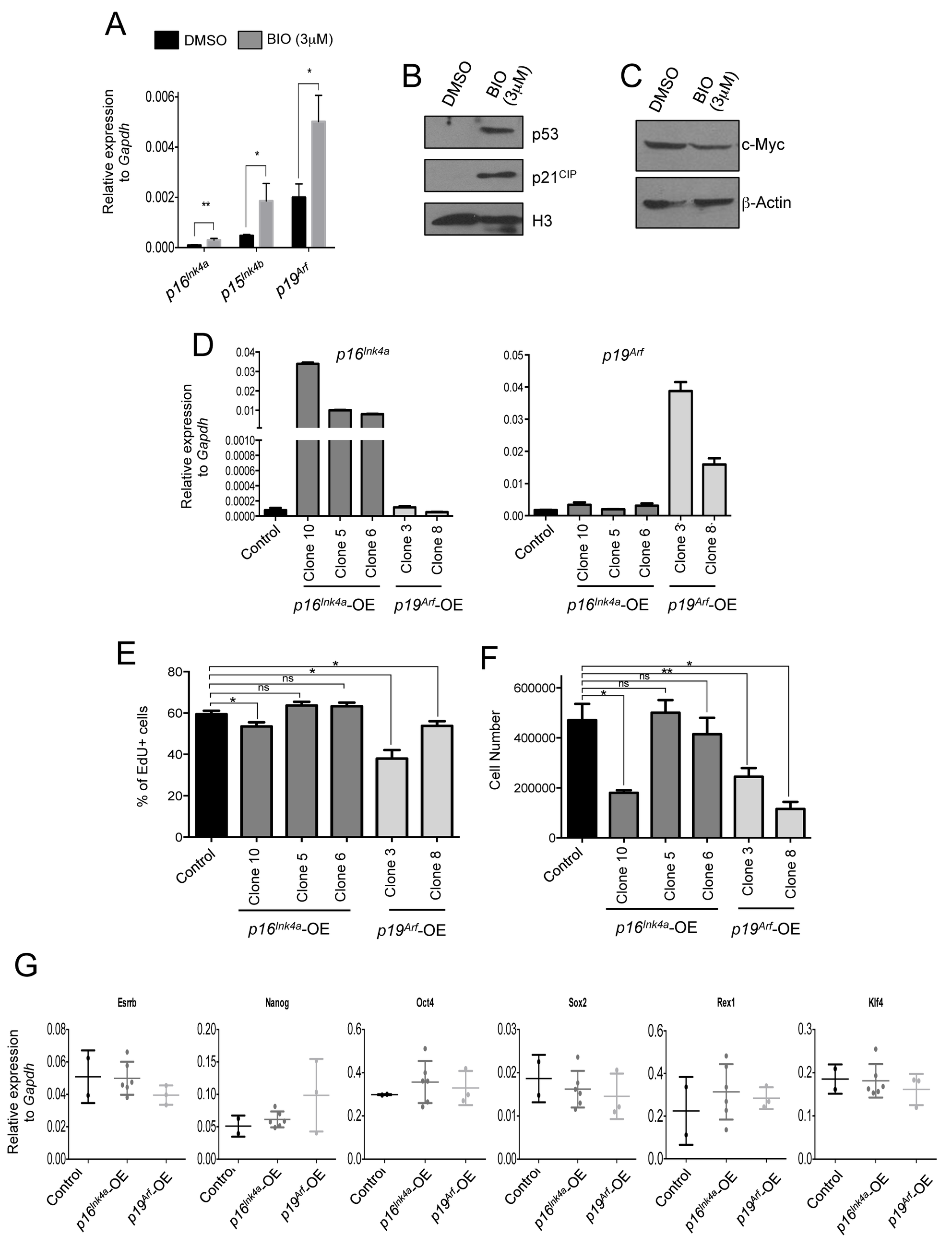

Supplement: S2 Fig — (A) qRT-PCR for Tcf1-recruited cell cycle genes (p15Ink4b, p16Ink4a and p19Arf) in control and BIO treated mESCs for 48h (n = 3). Results are presented as relative expression to Gapdh in order to visualize the expression levels of each transcript. (B) Representative Western blot of nuclear extracts of p53, p21Cip and Histone 3 (H3) as loading control in 6 days BIO treated WT mESCs. (C) Representative Western blot of total c-Myc and β-actin in control and BIO treated mESCs. (D) qRT-PCR of p16Ink4a and p19Arf in mESCs single clones infected for specific overexpression of p16Ink4a and p19Arf. Control cells were infected with empty vector. The clones are generated from a second independent infection corresponding to Fig 2C–2E. (E) Quantitative representation of number of EdU positive cells (EdU+) in control, p16Ink4a and p19Arf overexpressing mESCs (p16Ink4-OE, p19Arf-OE) 36h after plating (n = 3). Cells were incubated 40’ with EdU before fixation. (F) Cell counting quantification of control, p16Ink4a-OE, p19Arf-OE mESCs 48h after plating (n = 3). (G) qRT-PCR for pluripotent stem cell markers (Esrrb, Nanog, Oct4, Sox2, Rex1 and Klf4) in control (2 clones), p16Ink4a-OE (6 clones) and p19Arf-OE (3 clones) mESCs. All pooled data are represented as means ± SD. The asterisks indicate statistical significance by two-tailed Student’s t-test analysis (n.s. not significant; * p<0.05; ** p<0.01; ***p<0.001). (TIF) [file pgen.1006682.s002.tif]

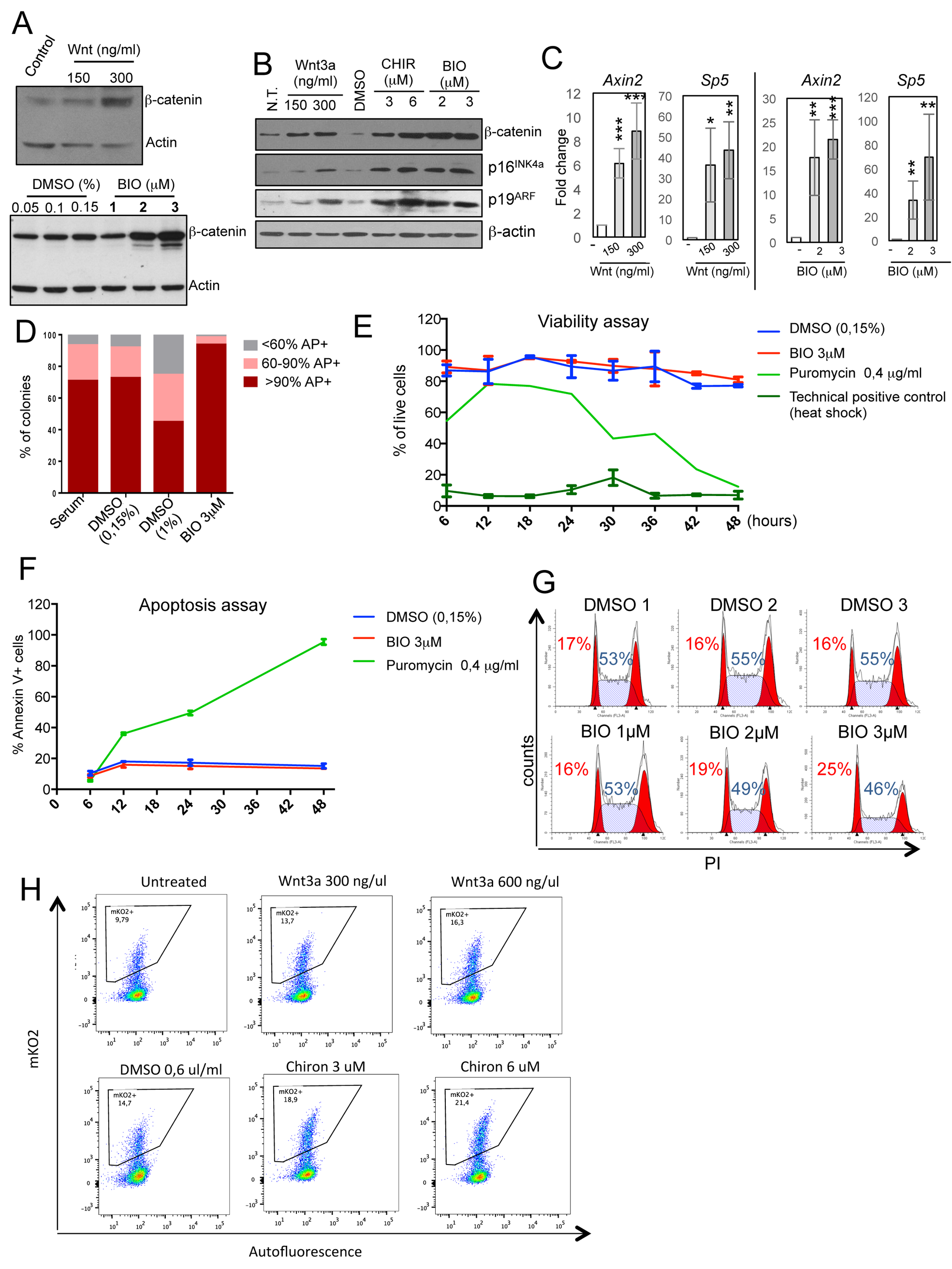

Supplement: S3 Fig — (A) Representative Western blots of total β-catenin and β-actin in mESCs treated with Wnt3a or BIO at indicated concentrations for 48h. (B) Representative Western blots of total β-catenin, p16Ink4a, p19Arf and β-actin in mESCs treated with Wnt3a, CHIR99021 and BIO for 48h at the indicated concentrations. (C) qRT-PCR for Wnt target genes (Axin2, Sp5) in Wnt3a or BIO treated mESCs at indicated concentrations for 48h (n = 4). (D) Quantitative representation of the number of colonies stained for Alkaline Phosphatase (AP) in untreated, DMSO and BIO treated mESCs. (E) Quantitative representation of live cells by FACS viability assay in time course of DMSO and BIO treated cells (n = 3; mean± S.E.M.). Puromycin was used as experimental positive control of cell death. For positive technical control of cell death, cells were treated with heat shock for 15’. (F) Quantitative representation of Annexin V positive (AnnexinV+) mESCs treated with indicated concentrations of BIO or DMSO for 6, 12, 24 and 48h. Puromycin was used as experimental positive control of cell death. (G) Representative cell cycle FACS analysis of propidium iodide stained mESCs treated with the indicated BIO concentrations for 72h. (H) Representative FACS analysis of mKO2-hCdt1 mESCs treated with indicated concentrations of Wnt3a and CHIR99021 for 72h. All pooled data are represented as means ± SD unless specifically indicated. The asterisks indicate statistical significance by two-tailed Student’s t-test analysis (* p<0.05; ** p<0.01; ***p<0.001). (TIF) [file pgen.1006682.s003.tif]

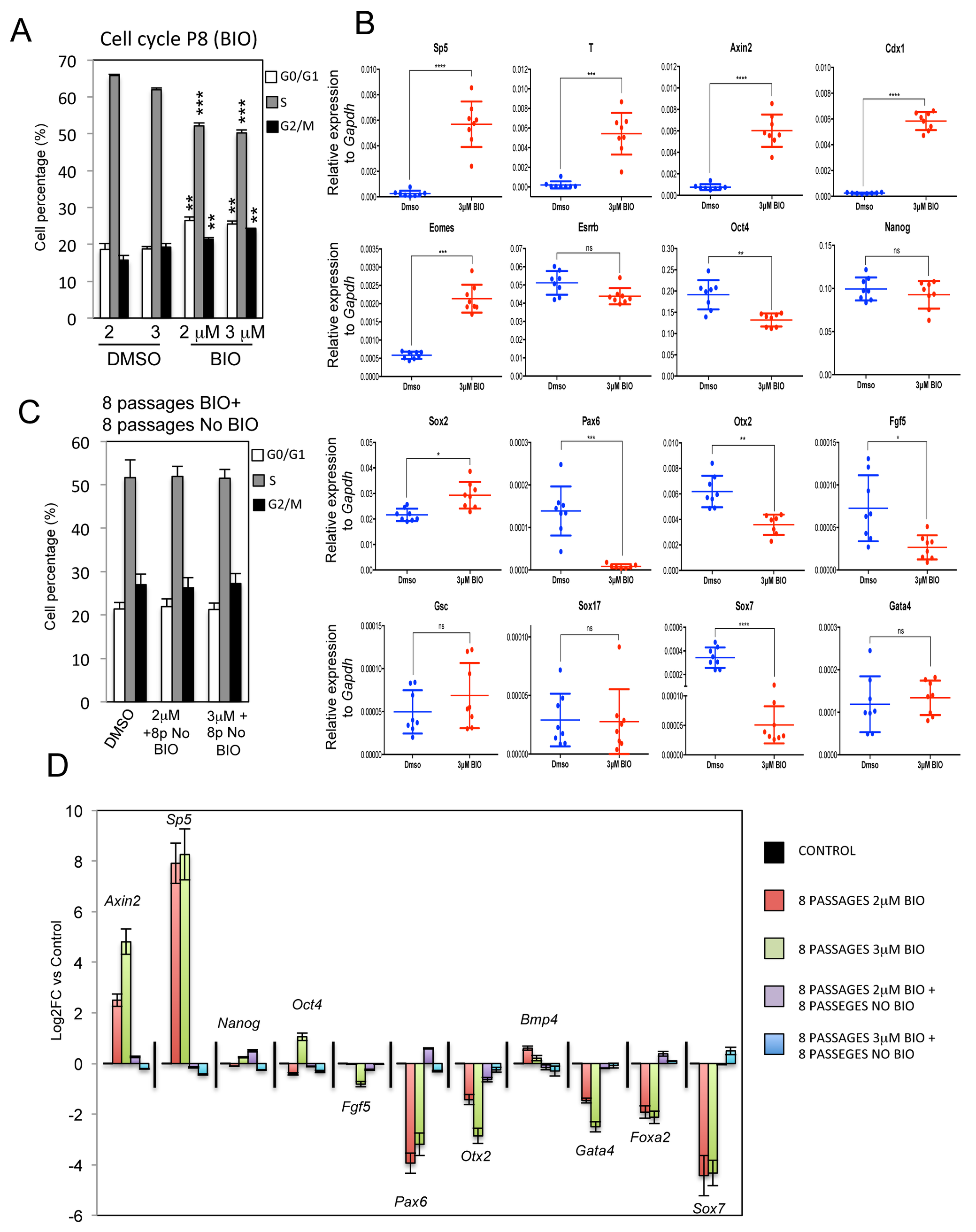

Supplement: S4 Fig — (A) Cell cycle quantification by FACS analysis of propidium iodide stained mESCs treated for 8 passages with BIO or with DMSO (n = 3; BIO-treated compared to DMSO-treated mESCs). (B) qRT-PCR for Wnt targets (Axin2, Sp5, T, Cdx1, Eomes), stem cell (Nanog, Oct4, Sox2, Esrrb), ectoderm (Fgf5, Pax6, Otx2), mesoderm (Gossecoid) endoderm (Gata4, Sox17 and Sox7) marker genes in independent mESCs clones treated with DMSO (0,15%) and BIO (3μM) for 8 passages. (C) Cell cycle quantification by FACS analysis of propidium iodide stained mESCs treated for 8 passages with BIO at the indicated concentrations + 8 passages in serum+LIF without BIO. (D) qRT-PCR of Wnt targets (Axin2, Sp5), stem cell (Nanog, Oct4), ectoderm (Fgf5, Pax6, Otx2), mesoderm (Bmp4) and endoderm (Gata4, Foxa2 and Sox7) genes in mESCs treated for 8 passages with BIO followed or not by additional 8 passages in serum+LIF medium without BIO. All pooled data are represented as means ± SD. The asterisks indicate statistical significance by two-tailed Student’s t-test analysis (* p<0.05; ** p<0.01; ***p<0.001). (TIF) [file pgen.1006682.s004.tif]

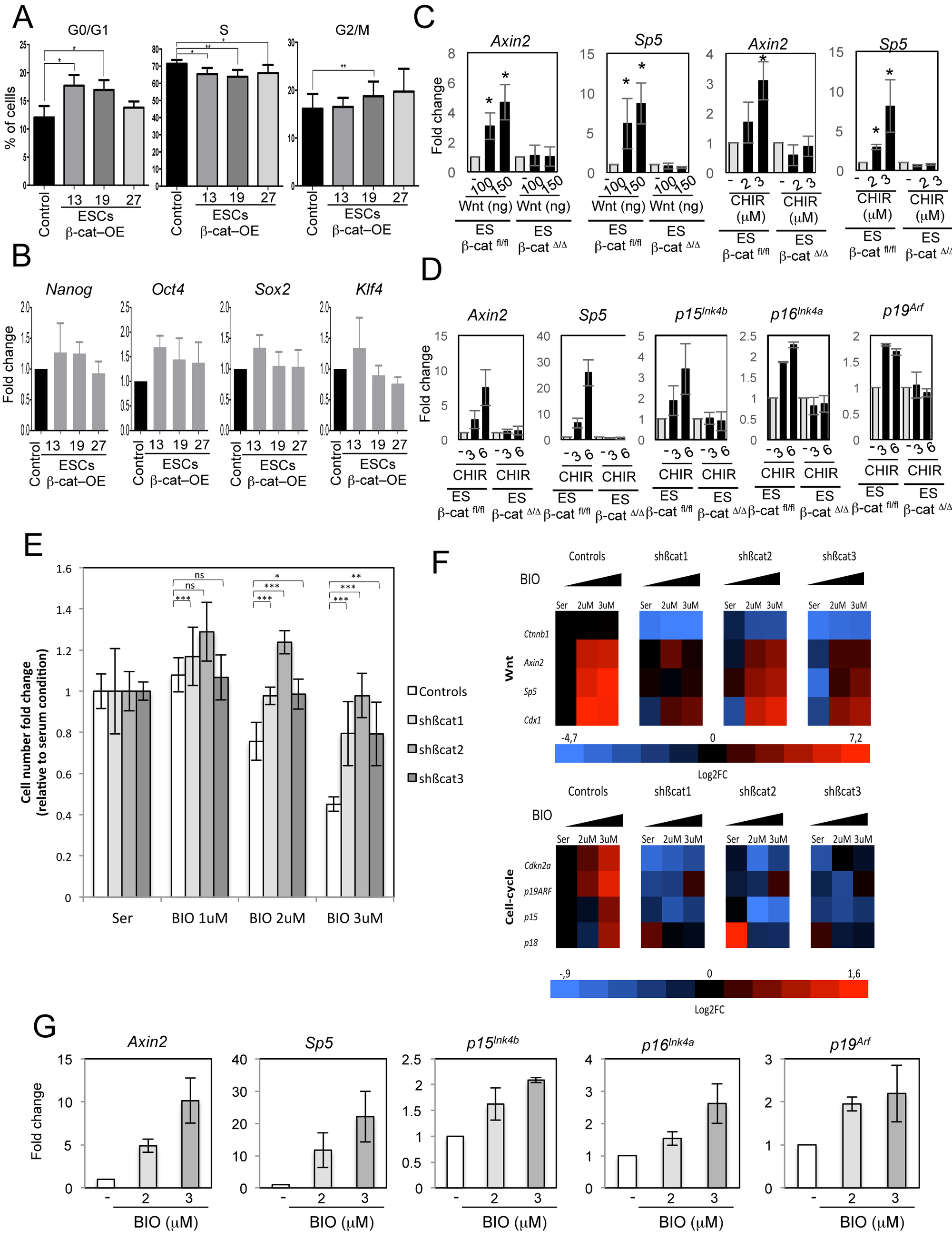

Supplement: S5 Fig — (A) Cell cycle FACS analysis after propidium iodide and EdU staining of control and ESCs-β-cat–OE clones (n = 4). (B) qRT-PCR of pluripotent stem cell markers in control and ESCs-β-cat–OE clones (n = 3). (C) qRT-PCR for Wnt targets (Axin2, Sp5) in control (β-cateninfl/fl) and β-catenin KO (β-cateninΔ/Δ) mESCs treated with Wnt3a or CHIR99021 at indicated concentrations (n = 3; treated cells compared to respective DMSO-treated mESCs). (D) qRT-PCR of cell cycle and Wnt targets in control (β-cateninfl/fl) and β-catenin KO (β-cateninΔ/Δ) mESCs treated for 48h at the indicated CHIR99021 (μM) concentrations (n = 2). (E) Cell number quantification of control mESCs and of three different pools of β-catenin silenced (shβcat) mESCs treated for 72h with indicated BIO concentrations (n = 3). (F) Heat map of representative qRT-PCR experiments for β-catenin (Ctnnb1), Wnt targets (Axin2, Sp5, Cdx1), and Tcf1 binding cell cycle genes (p15Ink4b, Cdkn2a, p18Ink4c and p19Arf) in control and in three different β-catenin silenced mESC pools (shβcat pool 1, 2 and 3) treated for 48h at the indicated BIO concentrations. (G) qRT-PCR for Tcf1 binding cell cycle genes (p15Ink4b, p16Ink4a, p19Arf) in untreated or BIO-treated mESCs Tcf3-/- at the indicated concentrations for 48h (n = 2). All pooled data are represented as means ± SD. The asterisks indicate statistical significance by two-tailed Student’s t-test analysis (n.s. not significant; * p<0.05; ** p<0.01; ***p<0.001). (TIF) [file pgen.1006682.s005.tif]

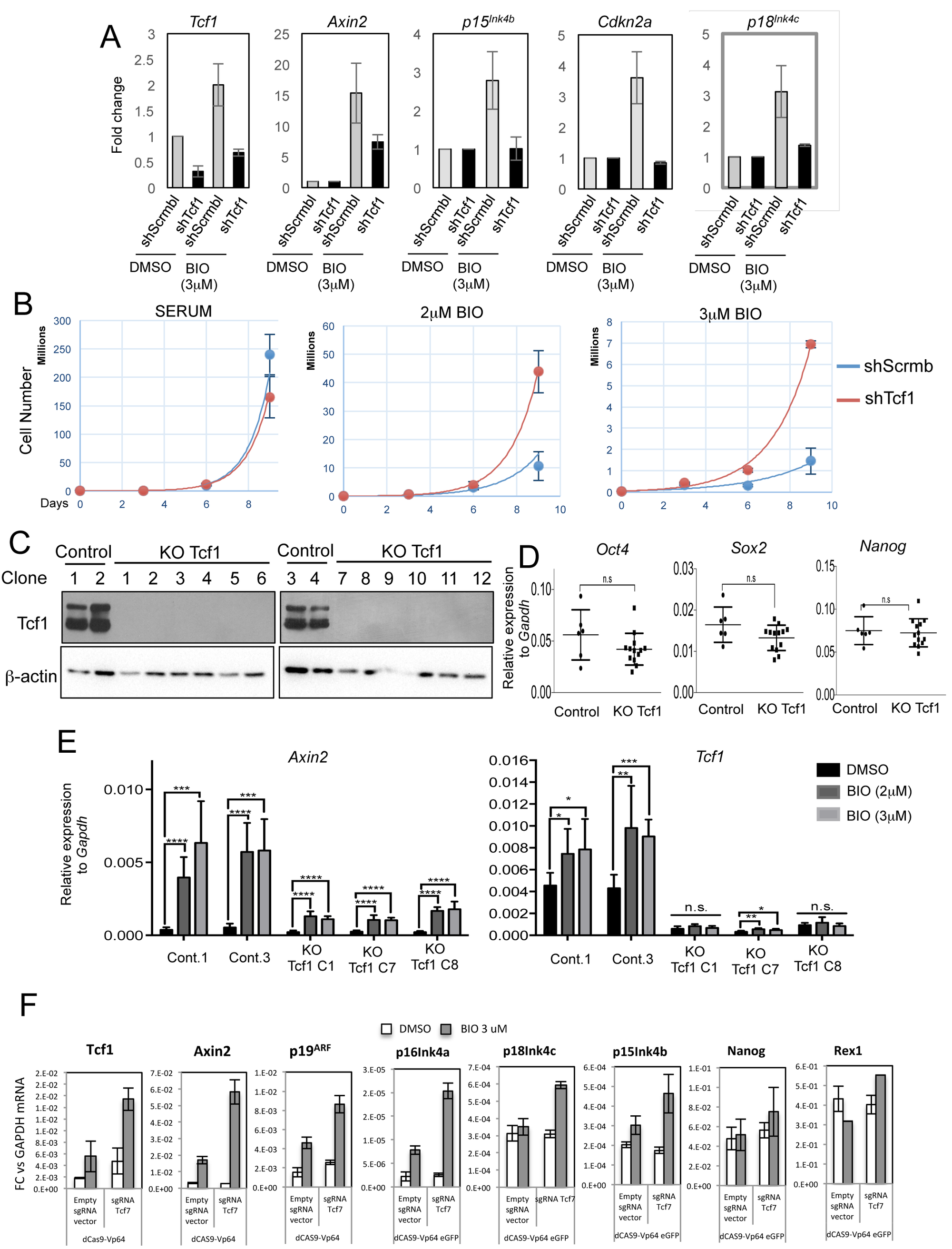

Supplement: S6 Fig — (A) qRT-PCR for Tcf1, Wnt target (Axin2) and Tcf1 binding cell cycle genes (p15Ink4b, p18Ink4c, Cdkn2a) in shScrmbl and shTcf1 mESCs treated at the indicated BIO concentration for 48h (n = 2). (B) Growth curve of shScrmbl and shTcf1 mESCs cultured for 3 passages and treated with the indicated concentrations of BIO (n = 2). (C) Representative Western blot of Tcf1 and β-actin in control and KO Tcf1 mESC clones generated by CRISPR/Cas9. (D) qRT-PCR for pluripotent markers (Oct4, Sox2 and Nanog) in control (6 mESCs clones) and KO-Tcf1 (13 mESCs clones). (E) qRT-PCR for Wnt target genes (Axin2 and Tcf1) in control and KO Tcf1 clones treated with BIO for 48h (n = 6; BIO-treated compared to respective DMSO-treated mESCs). (F) qRT-PCR for stem cell (Nanog, Rex1), Wnt targets (Tcf1 and Axin2) and Tcf1 cell cycle target genes (p15Ink4b, p16Ink4a, p18Ink4c, p19Arf) in control and Tcf1 overexpressing pool (sgRNATcf7) (one representative experiment). All pooled data are represented as means ± SD. The asterisks indicate statistical significance by two-tailed Student’s t-test analysis (n.s. not significant; * p<0.05; ** p<0.01; ***p<0.001). (TIF) [file pgen.1006682.s006.tif]

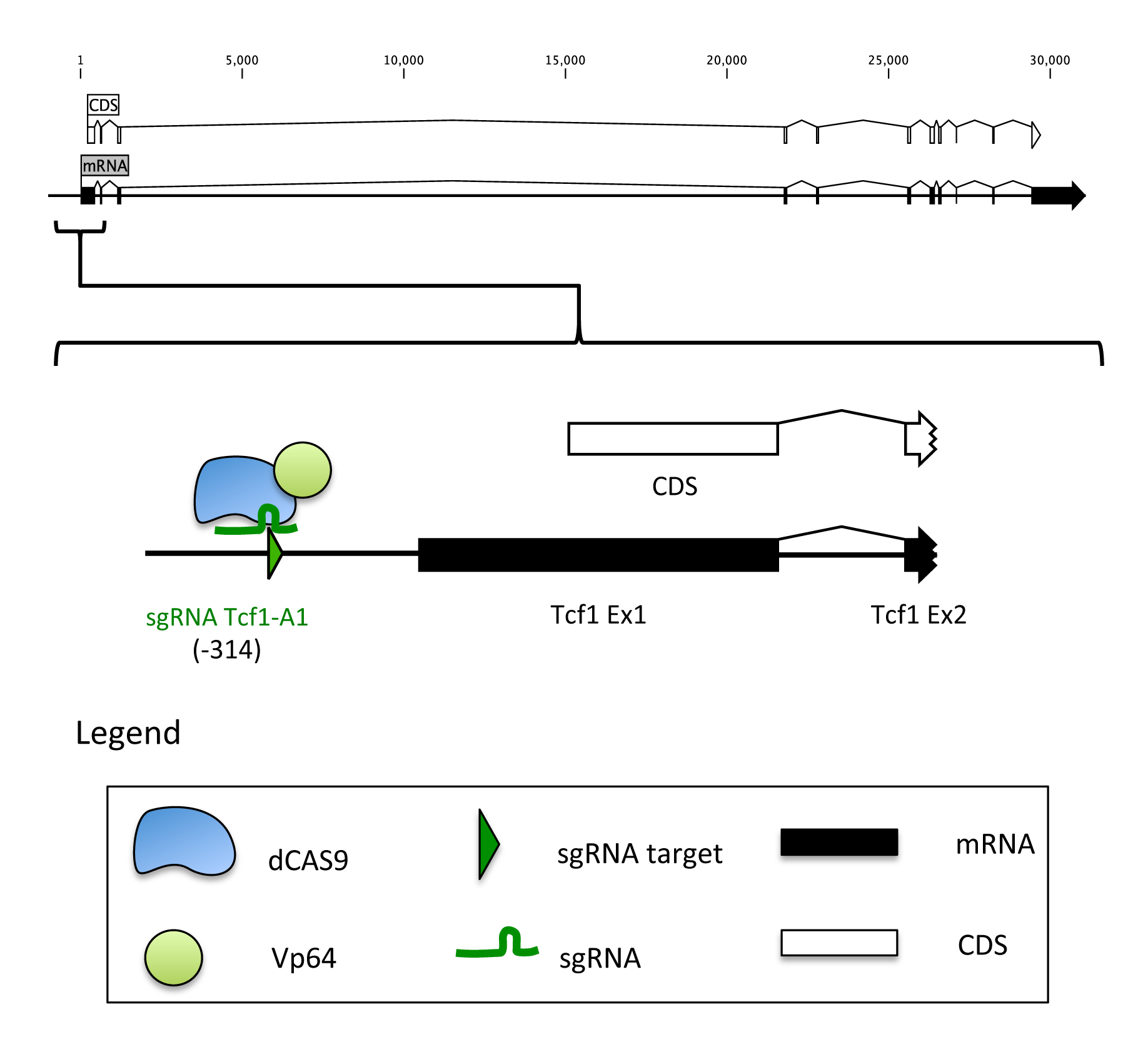

Supplement: S7 Fig — Schematic view of CRISPR/dCas9 method used to overexpress endogenous Tcf1. Two different sgRNAs targeting Tcf1 promoter region (108 and 314 bp from TSS of Tcf1) were used to allow binding of Cas9 fused with Vp64 transactivator domain to Tcf1 promoter in order to increase Tcf1 endogenous expression. (TIF) [file pgen.1006682.s007.tif]
